# Anatomical zone and tissue type impacts the repeatability of quantitative MRI parameters and radiomic features for longitudinal monitoring of treatment response in the prostate

**DOI:** 10.1007/s10334-025-01231-9

**Published:** 2025-02-22

**Authors:** Yu-Feng Wang, Sirisha Tadimalla, Lois Holloway, Niluja Thiruthaneeswaran, Annette Haworth

**Affiliations:** 1 Institute of Medical Physics, School of Physics, Faculty of Science, The University of Sydney, Sydney, NSW Australia; 2 Ingham Institute for Applied Medical Research, Liverpool, NSW Australia; 3 Sydney West Radiation Oncology, Westmead Hospital, Wentworthville, NSW Australia; 4 Liverpool and Macarthur Cancer Therapy Centre, Liverpool Hospital, Liverpool, NSW Australia; 5 South Western Sydney Clinical School, University of New South Wales, Liverpool, NSW Australia

**Keywords:** Magnetic resonance imaging, Repeatability, Prostate cancer, Quantitative parameter mapping, Voxel-based

## Abstract

**Objective:**

To (1) establish the repeatability coefficient (%RC) of region of interest (ROI) and voxel-wise measurements of a comprehensive range of quantitative MRI (qMRI) parameters and radiomic features in the prostate, and (2) assess the impact of different tissue types (benign vs tumor) and anatomical zones (peripheral, PZ, and non-peripheral, nPZ) on the %RCs.

**Methods:**

Test–retest qMRI was acquired in ten prostate cancer patients and six healthy volunteers. Parametric maps of apparent diffusion coefficient (ADC), diffusion coefficient (*D*), perfusion fraction (*f*), hypoxia score (HS), longitudinal relaxation time (T1), and observed transverse relaxation rate (R2*) were calculated. Fifty-nine radiomic feature maps were calculated from each of the parametric maps and T2-weighted images. The %RCs between tissue type and anatomical zones were compared using the Student’s t test at 95% significance level.

**Results:**

The %RC of ADC, *D* and HS, and up to 118 (out of all 413) radiomic features was significantly different between either anatomical zones, or between tumor and benign tissue, or both.

**Conclusions:**

DWI-derived parameters and a portion of their radiomic features require %RCs to be established specifically for anatomical zones, tumor and benign tissues. The remaining qMRI parameters and features can have a single threshold for the whole prostate.

**Supplementary Information:**

The online version contains supplementary material available at 10.1007/s10334-025-01231-9.

## Introduction

Quantitative magnetic resonance imaging (qMRI) parameters have shown promise in developing predictive machine learning models for non-invasive assessment and monitoring of response of prostate cancer (PCa) during and after radiation therapy (RT) [1]. Among the widely studied parameters are the apparent diffusion coefficient (ADC), derived from diffusion-weighted imaging (DWI), and the volume transfer constant (K^trans^) derived from dynamic contrast-enhanced (DCE) MRI. These parameters and many others, such as the transverse relaxation rate (R2*), and intra-voxel incoherent motion (IVIM) modeling parameters including diffusion coefficient (*D*), perfusion fraction (*f*), represent different biological characteristics, such as angiogenesis, cell density, aggressiveness, hypoxia and tumor grade [2–5]. Changes in these parameters during and after RT offer an opportunity to differentiate between acute effects, such as edema and long-term (desirable) effects related to cell death. Furthermore, radiomics features derived from qMRI parameter maps have demonstrated potential to improve accuracy in predicting PCa characteristics [6–8]. Thus, changes in these qMRI parameters and their derived radiomic features during and after RT are potential quantitative imaging biomarkers (QIBs) of treatment response. The purpose of these QIBs is to guide personalized clinical care, for example through either dose intensification to radioresistant tumor sub-volumes, focal therapy alone or the decision to use androgen deprivation therapy [9–13].

Longitudinal qMRI studies of treatment response biomarkers in PCa have typically relied on population-based statistical tests to assess changes in mean parameter values across a patient cohort relative to a pre-treatment baseline [8, 14–23]. However, a change in parameter value that is statistically significantly different from zero does not necessarily indicate a true treatment response, especially if the measurement error associated with the parameter is large [24]. Therefore, knowledge of measurement errors is essential to enable the utilization of QIBs for monitoring treatment response in individual patients. For example, although statistically significant increases in tumor ADC during RT (on the order of 10–40%) have been reported [16, 20, 21, 25], other studies have also shown that measurement errors can be as large as 42% [26]. The reliability of qMRI parameters and radiomic features in differentiating treatment-related changes from measurement uncertainties remains largely unknown in current literature, presenting a barrier for their practical application as QIBs for PCa response assessment.

In vivo test–retest imaging offers a means to yield reliable estimates of measurement uncertainties, considering both scanner-related and day-to-day biological variations. The Quantitative Imaging Biomarker Alliance (QIBA) has recommended the use of the repeatability coefficient (%RC or RC) to quantify test–retest repeatability, representing the minimum detectable difference between two repeated measurements under identical conditions [27, 28]. Therefore, the %RC or RC can be used to define the threshold of minimum change required in qMRI parameters and radiomic features in longitudinal studies to differentiate true treatment response from measurement uncertainty.

Several studies have reported the test–retest repeatability of prostate ADC measurements [26, 29–32], with %RC values of 30% for ADC calculated over the entire gland [26]. When separated based on anatomical zone, %RC values of up to 30 and 15% were observed in the peripheral zone (PZ) and non-peripheral zone (nPZ), respectively [26, 29, 30]. Other studies have reported %RC values of ADC of up to 42 and 30% in tumor and benign prostate, respectively [26, 30–32]. A few studies have reported %RC values for prostate K^trans^ [33, 34]; however, repeatability of other qMRI parameters in the prostate such as R2* remains unknown. Likewise, while studies have analyzed the repeatability (reporting metrics such as intra-class correlation coefficients) of radiomic features derived from T2w images and ADC maps [35–37], quantification of %RC has been limited to features derived from T2w images [37]. There is little to no information on the repeatability of these parameters in the different anatomical zones and tissue types. The observed differences in %RC in DWI parameters between anatomical zones and tissue types are likely due to spatially varying imaging errors, such as susceptibility and motion artifacts. As some qMRI methods are more sensitive than others to these artifacts, it is reasonable to expect a varying impact of anatomical zone and tissue type on the repeatability of different qMRI parameters and their associated radiomic features.

Additionally, the majority of the prostate qMRI repeatability metrics thus far reported in the literature rely on a region of interest (ROI)-based approach, calculating the %RC of an average measurement over an entire ROI, which could be the whole-gland, an anatomical zone, tumor, or benign tissue region. While these repeatability measurements are well suited to help interpret longitudinal changes in average parameter values in these ROIs, it is known that PCa is not only a heterogenous disease, but its response to radiation is also heterogenous [10, 38]. Prediction of tumor progression using voxel-wise analysis of longitudinal qMRI data has been reported in non-prostate cancers [39–41] and is a growing area of interest in real-time RT adaptation and biological optimization of RT [9, 42–44]. However, uncertainties in qMRI parameter and radiomics feature estimation are likely to be larger in individual voxel measurements due to factors, such as low SNR, test–retest image registration errors, and spatial signal variance due to image artifacts [45–47].

The aim of this study was to determine the %RC for both ROI and voxel-wise measurements of an extensive range of qMRI parameters and radiomic features derived from T2w images, DWI, R2* and T1 maps. The differences in the repeatability of qMRI parameter and radiomic features between tissue types (tumor vs benign tissue) and anatomical regions were tested to determine the need to establish %RCs separately for each region.

## Methods

### Participant information

Test–retest MRI scans were acquired in two cohorts of participants. The first cohort consisted of patients diagnosed with PCa and imaged at two time points, one to two weeks apart, prior to RT. The second cohort consisted of healthy volunteers who were imaged at variable time points within a period of 12 weeks.

#### Patients

A cohort of 10 patients were recruited to the SI-BiRT-2 study between June 2020 and June 2021. This study received Human Research Ethics Committee (HREC) approval, and patients were imaged and treated at the Crown Princess Mary Cancer Centre, Westmead Hospital, Australia (HREC: 2019/ETH10022, ANZ U1111-1221-9589). Inclusion criteria were patients with biopsy proven localized PCa who will receive RT without neoadjuvant hormone therapy. Patients were excluded if they had any previous hormonal or surgical treatments, or prior pelvic RT. Baseline characteristics of the patients are summarized in Table 1. Test–retest MRI scans, one to two weeks apart, were performed for each patient prior to RT. Fiducial markers (in all 10 patients) and hydrogel spacer (in 9 patients) insertion were performed 11–26 days prior to the first scan to reduce the impact of hemorrhage in the prostate on MRI.

**Table 1:** Median (range) of age and pre-treatment prostate specific antigen (PSA) level and the number of patients with the Gleason grade group

Number of patients	10
Age	69.5 (62–79) years
PSA	8.4 (3.8–11.1) ng/ml
Gleason Grade Group	
2	8
3	2
T stage	
T1c	8
T2	2

#### Volunteers

Six male volunteers, with no known prostate disease, participated in a prospective study approved by the local Institutional Review Board at Liverpool Hospital, Liverpool, Australia (HREC: 2019/ETH04295). Inclusion criteria were healthy male, > 18 years old, no contraindication to MRI, and no significant medical history of relevance to this study. The median age of the volunteer cohort was 33.5 years (range 29–41).

### MRI data acquisition

Patient MRI data were acquired on a 3.0 T MAGNETOM Prisma scanner (Siemens Healthineers, Erlangen, Germany). The volunteer cohort MRI data were acquired on a 3.0 T MAGNETOM Skyra scanner (Siemens Healthineers, Erlangen, Germany). The MRI protocol consisted of imaging sequences recommended by the Prostate Imaging–Reporting and Data System (PI-RADS v2), including axial 2D T2w imaging and DWI. DCE-MRI was not explored in this study due to the requirement for repeated contrast injections. Additionally, an isotropic 3D T2w sequence was acquired in the sagittal plane to facilitate registration of the longitudinally acquired scans. DWI was performed with nine b-values (PI-RADS recommendation ≥ 2) ranging from 0 to 800 s/mm^2^ to enable the estimation of IVIM model parameters. Longitudinal relaxation time (T1) mapping was performed using a 3D variable flip angle (VFA) spoiled gradient echo (SPGR) sequence (VIBE, Siemens Healthineers, Erlangen, Germany) with 6 flip angles. Radiofrequency transmit field (B1^+^) maps were acquired to enable correction of errors in the T1 values arising from inhomogeneous B1^+^ fields. Finally, total transverse relaxation rate (R2*) mapping was performed with a multi-echo gradient echo (MEGRE) sequence. The acquisition parameters were matched where possible between the two scanners, with details of the imaging parameters are presented in Table 2 in the order of acquisition (left to right). Endorectal coils and enemas were not used in consideration of participant comfort. Previously reported longitudinal measurements indicated that intra-scanner repeatability was similar for both scanners, with ADC, T1, and R2* measurements being repeatable within 2, 7, and 5%, respectively [48].

**Table 2:** Image acquisition parameters for axial and sagittal T2-weighted (T2w) imaging, diffusion-weighted imaging (DWI), T2* weighted multi-echo gradient echo (MEGRE) imaging, B1 field mapping, and T1 mapping

	2DT2w	DWI	R2* mapping	3DT2w	B1 + mapping	T1 mapping
Sequence	TSE	ZOOMit	MEGRE	SPACE	TFL	VFA SPGR
Plane	Axial	Axial	Axial	Sagittal	Axial	Axial
Mode	2D	2D	2D	3D	2D	3D
TR (ms)	> 7000	> 4000	810	> 1600	> 8500	4.63
TE (ms)	93	60 (Prisma) and 70 (Skyra)	4.92–73.8 (12 echoes)	105 (Prisma) and 104 (Skyra)	2	1.38
Flip angle (°)	160	180	40	120	8	2/5/10/ 15/20/30
Matrix size	384 × 384	100 × 56	128 × 128	256 × 256	64 × 52	224 × 182
FOV (mm)	200 × 200	190 × 105.6	220 × 220	200 × 200	200 × 162.5	250 × 203.1
Slice thickness (mm)	2.5	4	4	0.8	5	3
In-plane resolution (mm^2^)	0.5 × 0.5	1.9 × 1.9	1.7 × 1.7	0.8 × 0.8	3.9 × 3.9	1.1 × 1.1
Other parameters		*b* = 0, 20, 50, 100, 200, 300, 400, 600, 800 s/mm^2^ ≥ 3 directions (averages of 1, 1, 2, 2, 3, 3, 4, 4, 7, respectively)				
Scan time (approx.)	5:30	5 (Prisma) and 8 (Skyra) min	5:34	7:36	0:08	2:44

*TSE* turbo spin echo, *ZOOMit* reduced field of view echo-planar imaging (EPI), *MEGRE* multi-echo gradient echo imaging, *SPACE* 3D volume T2w turbo spin echo sequence, *TFL* Turbo FLASH, *VFA SPGR* variable flip angle spoiled gradient echo, *TE* echo time, *TR* repetition time, *FOV* field of view

### Image processing

The 2D axial and 3D sagittal T2w images were corrected for the bias field effect using the N4ITK algorithm implemented in the *N4ITKBiasFieldCorrections* module in 3D Slicer (version 4.10.2) [49]. A linearized mono-exponential model was applied to DWI with b-values 50 and 800 s/mm^2^ to obtain maps of apparent diffusion coefficient (ADC). These values were selected based on those commonly used in clinical practice [50]. The simplified intra-voxel incoherent motion (IVIM) model proposed by Hompland et al. [5] (assuming negligible impact of intravascular pseudo-diffusion on signal loss at b-values > 200 s/mm^2^) was applied to DWI to obtain maps of the diffusion coefficient (*D*) and the perfusion fraction (*f*). The hypoxia score (HS) was then calculated using the *D* and *f* maps, representing the consumption and supply of oxygen, respectively [5]. As the patient characteristics in our study were different to the HS model cohort, no scaling was applied to match the distributions of D and f prior to the calculation of HS [51]. Maps of R2* were obtained by fitting a mono-exponential model to the MEGRE data. The flip angle correction factor map was calculated by dividing the actual flip angle map (inline output from B1^+^ mapping) by the applied flip angle of 90°. T1 was estimated from the VFA data by fitting the SPGR signal model with the applied flip angles uniformly scaled by the flip angle correction factors [52].

### Radiomic feature extraction

Radiomic features were calculated at each voxel from the axial T2w images and all (six) qMRI parameter maps. To ensure consistency of parameter extraction, all images and maps were resampled to an isotropic resolution of 1 × 1 × 1 mm^3^. The images were normalized using the function *normalizeImage* in the pyradiomics library (version 3.0.1) with a scaling factor of 100, resulting in gray values ranging from − 300 to 300 [53]. Voxel-wise radiomic features were extracted in each image slice using a 5 × 5 mm sliding window and a bin width of 20. Fifty-nine radiomics feature maps from the gray level co-occurrence matrix (GLCM, *n* = 24), gray level run length matrix (GLRLM, *n* = 16), gray level dependence matrix (GLDM, *n* = 14), and the neighboring gray tone difference matrix (NGTDM, *n* = 5) classes were extracted from each image type using the pyradiomics library, compliant with the Imaging Biomarker Standardization Initiative [53, 54]. A total of 413 (7 image sets × 59 features) radiomics features were extracted, as detailed in Supplementary Data 3.

### Image registration

**Fig. 1:**
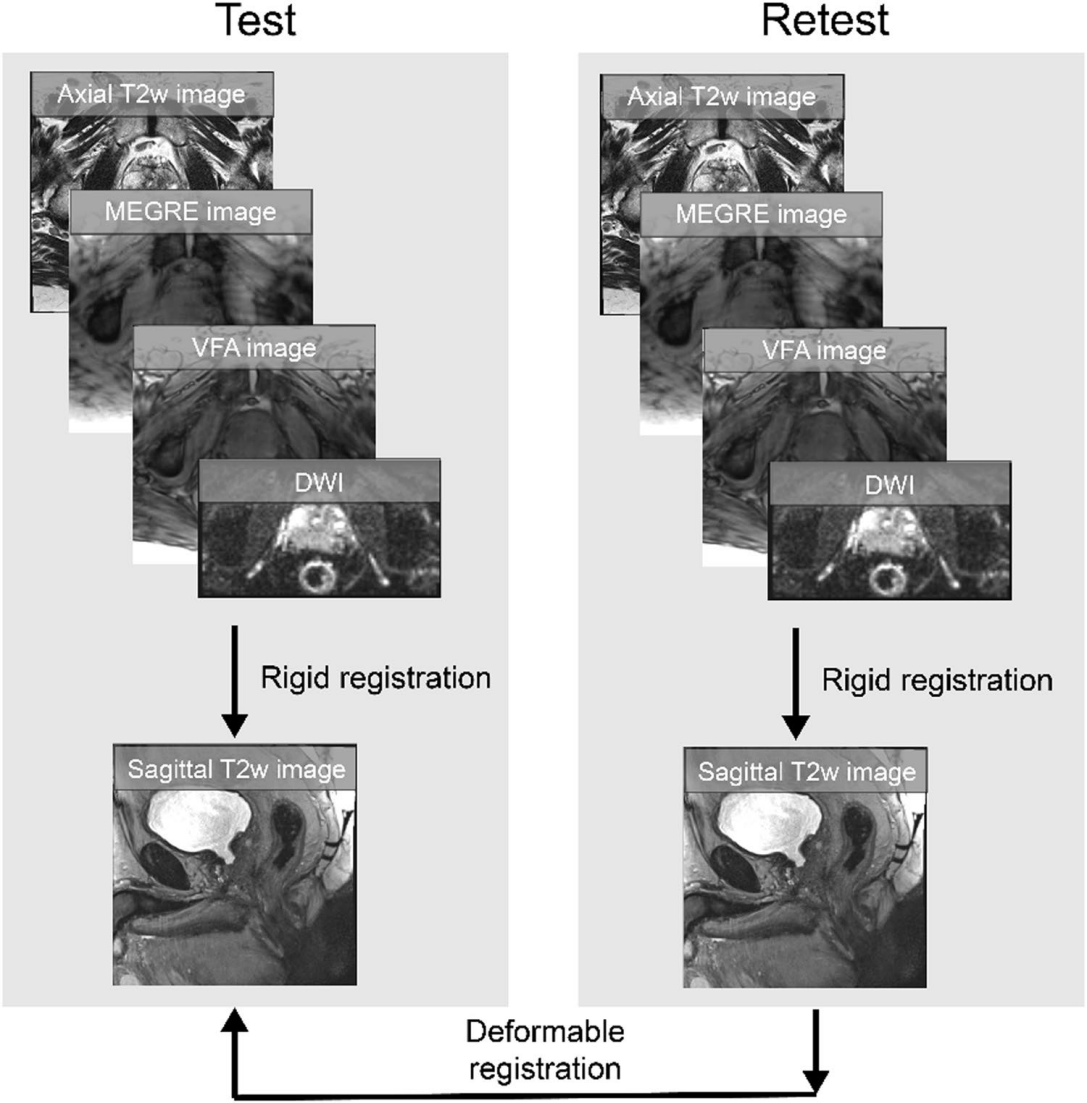
Image registration workflow of test–retest T2-weighted (T2w) images, multi-echo gradient echo (MEGRE) images, variable flip angle (VFA) spoiled gradient echo images, and diffusion-weighted imaging (DWI)

To enable both ROI and voxel-wise analysis of the test–retest qMRI parameter and radiomics feature measurements, the axial T2w image, qMRI parameter maps and their radiomics feature maps from the repeated scan were deformably registered to the first sagittal T2w image baseline scan. The registration workflow is shown in Fig. 1. At each time point, the axial T2w images, DWI and relaxation time mapping images were rigidly registered to the sagittal T2w images, and the corresponding transformation matrix was then applied to the qMRI parameter maps and their radiomic features. The sagittal T2w images acquired at the retest scan were deformably registered to the sagittal T2w images acquired at the first scan. The corresponding transformation matrix was then applied to the axial T2w images, qMRI parameters and their radiomic features resulting in all imaging data being re-sampled into the baseline scan reference space. Details of the registration are summarized in Supplementary Methods. Registered images were visually assessed for alignment of the prostate gland, PZ and urethra. Sixty percent of the rigid registrations between the axial T2w image, DWI, and relaxation time mapping images with the sagittal T2w images required minor manual adjustments by less than 5 mm to improve the alignment. All deformable registrations between the test–retest sagittal T2w images were considered “acceptable” with manual adjustments providing no improvement in image alignment.

### Region of interest delineation

The whole prostate gland (WG) and peripheral zone (PZ) were automatically segmented using an open-source deep learning model [55]. The model generated segmentations on the sagittal T2w image and uses both the sagittal T2w image and the axial T2w image (resampled to the sagittal T2w space) as input. The WG and PZ segmentations were visually inspected and edited manually when segmentations were incomplete or outside the prostate gland. The WG was eroded by 1 mm in 3D to reduce the risk of including non-prostate tissue due to contouring errors at the periphery of the prostate. The nPZ was defined by subtracting the PZ from the WG. Tumor regions were automatically segmented using an inhouse developed deep learning model [56]. The output of the deep learning model is a probability map, i.e., the image intensity in each voxel is the probability of the presence of tumor in the voxel. The tumor mask was obtained by applying automatic histogram-based multi-level thresholding on the probability maps using the Yen’s algorithm implemented in scikit-image [57].

### Data analysis

#### ROI measurement of qMRI parameters and radiomic features

For the ROI analysis, the median quantitative parameter value (qMRI parameters and radiomic features) of each tissue type (healthy tissue from volunteer data, and benign and tumor tissue from patient data) in each region (WG, PZ, nPZ) was calculated for each scan.

#### Repeatability of ROI and voxel-wise measurement of qMRI parameters and radiomic features

Based on our previous phantom study conducted on the same scanners [48], it was determined that the repeatability of ADC, T1 and R2* is dependent on the absolute parameter values. Therefore, we have applied the %RC instead of an absolute RC as recommended in the QIBA guidelines [27, 28]. To calculate %RC, the repeatability of the ROI and voxel-wise measurements were first assessed using the coefficient of variation (%CV) calculated as the ratio between the standard deviation and the mean of repeated measurements (Eq. 1). Lower %CVs represent higher repeatability.

1 $$\text{\%CV}=\frac{\text{standard deviation}}{\text{mean}}\times 100$$

The within-subject %CV (%wCV) was calculated from the root mean square of the %CV over N subjects to quantify the repeatability in concordance with guidelines established by the QIBA [27, 28]. For ROI analysis, the %wCV_ROI_ was calculated from the %CV_ROI_ of each subject (Eq. 2), resulting in one %wCV_ROI_ for the cohort. For voxel-wise analysis, the %wCV_voxel_ was computed from the %CV_voxel_ of each voxel within one subject (Eq. 3), resulting in one %wCV_voxel_ per subject in the cohort.

2 $${\text{\%wCV}}_{\text{ROI}}= \sqrt{\frac{\sum_{i=1}^{{N}_{\text{subjects}}}{\text{\%}{\text{CV}}_{\text{ROI}}}^{2}}{{N}_{\text{subjects}}}}$$

3 $${\text{\%wCV}}_{\text{voxel}}= \sqrt{\frac{\sum_{i=1}^{{N}_{\text{voxels}}}{\text{\%}{\text{CV}}_{\text{voxel}}}^{2}}{{N}_{\text{voxels}}}}$$

The repeatability coefficient (%RC), calculated as 2.77 times the %wCV, is an estimate of the 95% confidence interval of the measurement uncertainty. For ROI analysis, %RC (%RC_ROI_) was calculated using the %wCV_ROI_ (Eq. 4) [28]. For voxel-wise analysis, the %RC (%RC_voxel_) was calculated per subject using the %wCV_voxel_ (Eq. 5) [58].

4 $${\text{\%RC}}_{\text{ROI}}=2.77\times {\text{\%wCV}}_{\text{ROI}}$$

5 $${\text{\%RC}}_{\text{voxel},\text{subject}}=2.77\times {\text{\%wCV}}_{\text{voxel},\text{subject}}$$

#### Statistical analysis

The repeatability metrics (%CV_ROI_ and the %wCV_voxel_ for ROI and voxel-wise measurements, respectively) were compared across two sets of ROIs using a two-tailed paired *t* test. The first comparison examined the impact of the two anatomical zones (PZ and nPZ), using data from both patients and volunteers. The second comparison assessed the effect of tissue type (tumor and benign), and therefore, used only patient data. The *ttest_rel* function in the scipy.stats library was used [59], and all statistical tests were conducted at a significance level of 0.05.

#### Ranking of radiomic features

Radiomic features extracted from ADC, *D*, *f*, HS, R2* and T1 maps, and T2w images were pooled and ranked by their %RCs to obtain the top 50 most repeatable features. For voxel-wise analysis, the median %RC_voxel_ was used. In radiomic features with no dependence of repeatability on tissue type or anatomical region, the %RCs derived from the WG were used in the ranking. As radiomic features with dependence of repeatability on tissue type or anatomical region, or both, will have multiple %RCs, derived from each respective ROI, the largest %RC of each feature was used in the ranking. The 50 radiomic features with smallest %RCs were selected separately for ROI and voxel-wise measurement approaches.

## Results

**Fig. 2:**
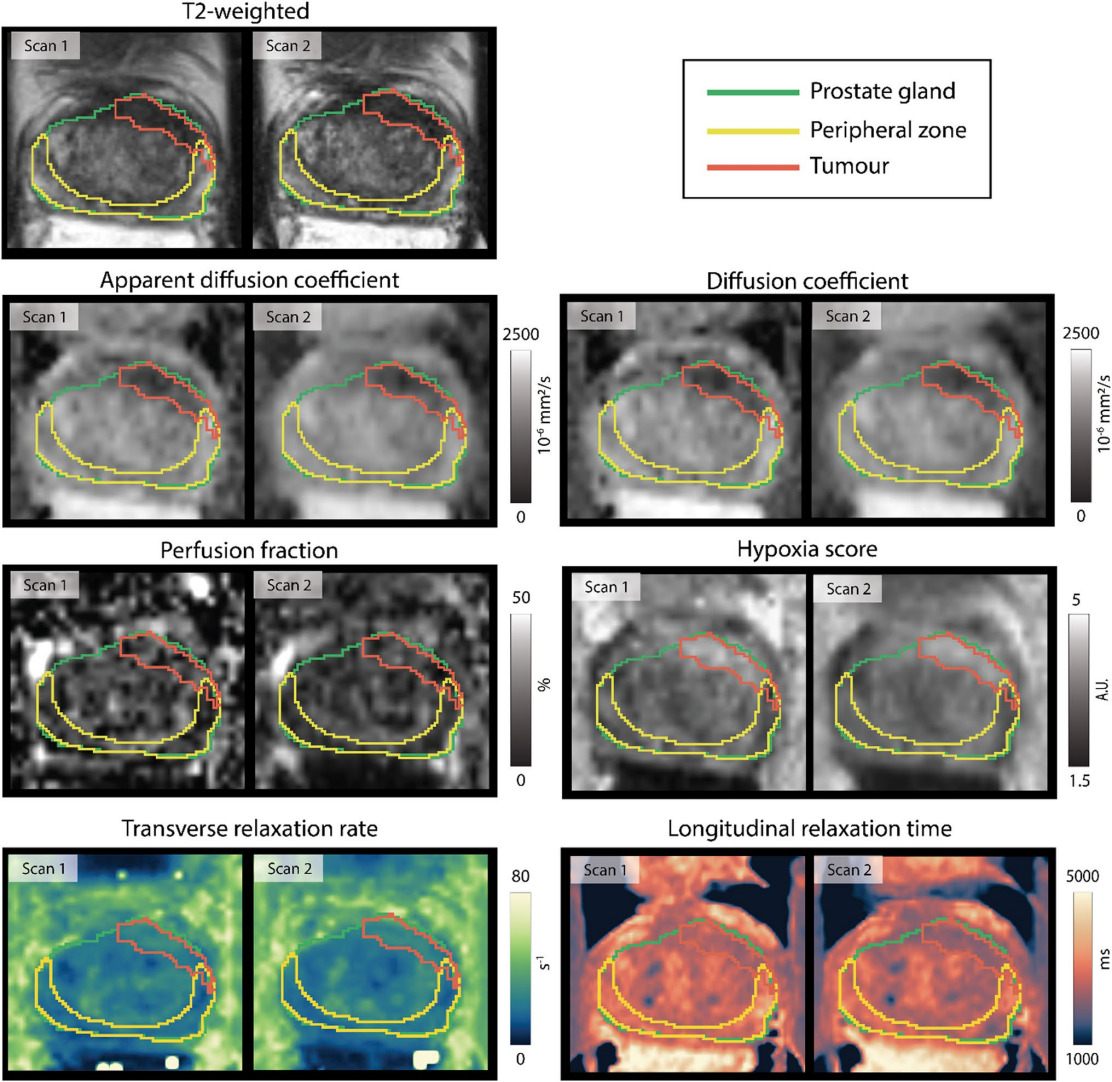
Example of the registered test–retest axial T2-weighted images, apparent diffusion coefficient, diffusion coefficient, perfusion fraction, hypoxia score, transverse relaxation rate, and longitudinal relaxation time maps of a patient. All images were registered to the sagittal T2-weighted image of the first scan. The prostate gland, peripheral zone, and tumor region of interests, delineated on the sagittal T2-weighted image of the first scan, were propagated to each qMRI parameter map and axial T2-weighted image

A total of 38 MRI datasets were acquired (18 from 6 volunteers and 20 from 10 patients). Datasets from two participants (one volunteer and one patient) were excluded from the analysis due to severe susceptibility artifacts and distortions caused by rectal gas. Hence, a total of 34 MRI datasets were analyzed for this study. The ADC, *D*, *f*, HS, R2* and T1 measurements are summarized in Supplementary Data 1. The registered test–retest qMRI parametric maps from one patient and one volunteer are shown in Fig. 2 and Supplementary Fig. 1, respectively.

### Repeatability of qMRI parameters

The repeatability of ROI and voxel-wise measurements of qMRI parameters in different tissue types (benign vs tumor) and anatomical regions (PZ vs. nPZ) are presented in Supplementary Data 2. Overall, HS was found to be the most repeatable qMRI parameter (%RC range of 3–12%). This is despite D and f (which are used in the calculation of HS) which showed higher %RCs (6–27% and 25–73%, respectively). The differences in %RC observed based on anatomical zone and tissue types are summarized below.

#### ROI measurements

The repeatability of ADC and *D* measurements was significantly different between tissue types (*p* < 0.05), with the %RC in tumor tissue being twice that observed in benign tissue. The repeatability of HS, *f*, R2* and T1 measurements was not found to be significantly different between tumor and benign tissue (*p* = 0.1–0.98).

The repeatability of ADC, *D*, and HS measurements was found to be significantly different between anatomical zones (*p* < 0.05), with %RC in the PZ two times higher than the nPZ. The repeatability of *f*, R2*, and T1 measurements was not found to be significantly different between anatomical zones (*p* = 0.12–0.62).

#### Voxel-wise measurements

The repeatability of voxel-wise ADC and *D* measurements was significantly different between tissue types (*p* < 0.05), with %RC in the tumor tissue double that measured in the benign tissue. The repeatability of HS, *f*, R2*, and T1 was not found to be significantly different between tissue types (*p* = 0.18–0.72). The repeatability of voxel-wise measurements of all qMRI parameters (ADC, *D*, HS, *f*, R2*, and T1) was not found to be significantly different between anatomical zones (*p* = 0.07–0.9).

%RC values for the qMRI parameters, according to their dependence on tissue type and anatomical region are given in Table 3.

**Table 3:** Summary of repeatability coefficients (%RC) for qMRI parameters according to dependence of measurement repeatability on anatomical zones (peripheral vs non-peripheral zone) and tissue types (benign vs tumor)

qMRI parameter	Tissue type	Analysis approach		
		Region of interest		Voxel-wise [median (range)]
A. Dependent on both anatomical zone and tissue type				
		PZ	nPZ	WG
ADC	Benign	15	6.4	29 (16–37)
	Tumor	29	13	43 (30–83)
*D*	Benign	16	6.5	28 (16–37)
	Tumor	27	11	38 (25–78)
B. Dependent on anatomical zone				
HS	All	7.9	3.2	19 (11–27)
C. Not dependent on anatomical zone or tissue type				
*f*	All	26	26	130 (95–160)
R2*		39	39	55 (27–120)
T1		10	10	25 (17–77)

*WG* whole gland, *PZ* peripheral zone, *nPZ* non-peripheral zone

### Repeatability of radiomic features

**Fig. 3:**
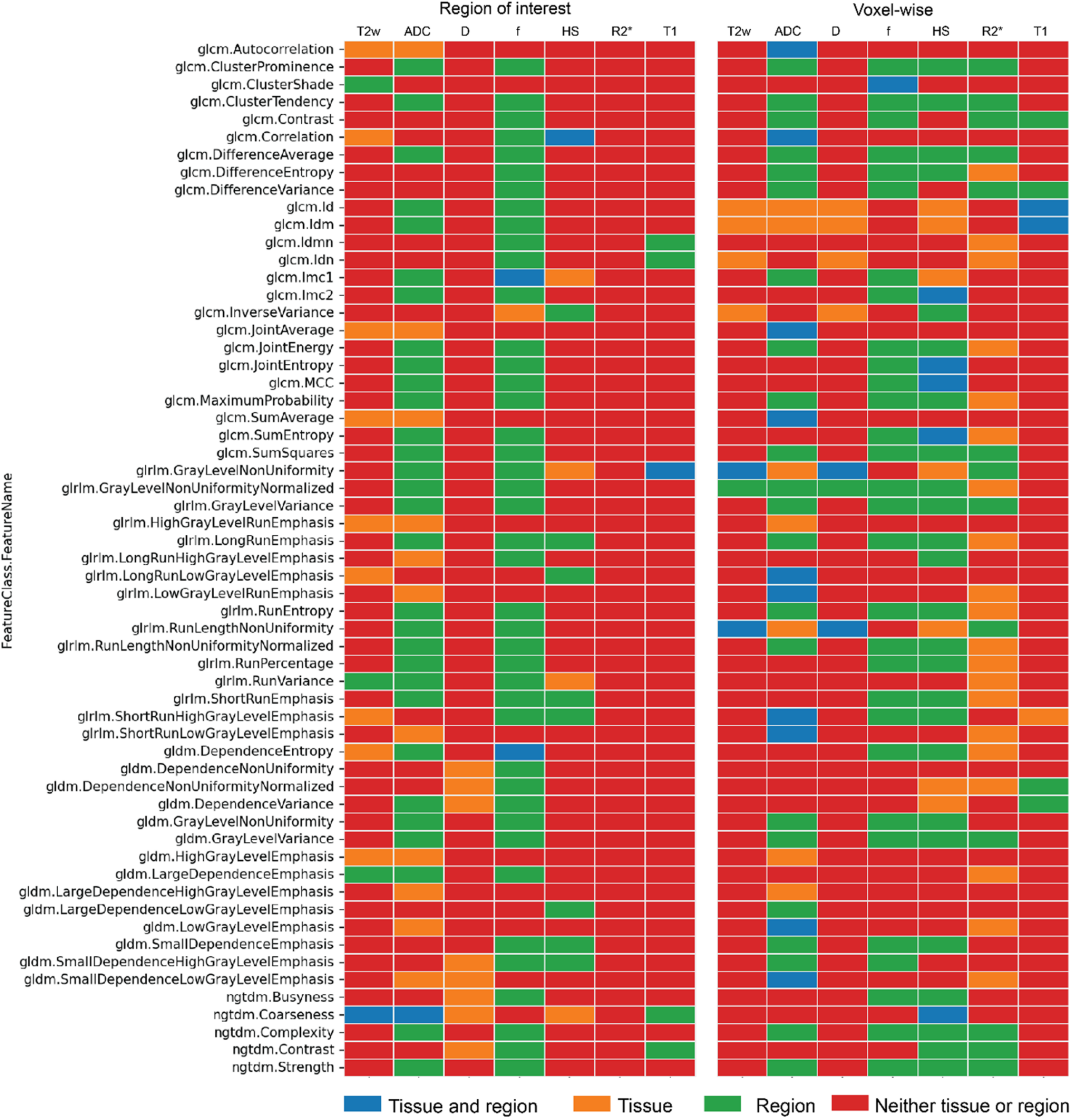
Summary of dependence of repeatability coefficients (%RCs) on tissue type and anatomical zone of radiomic features derived from T2w images, ADC, *D*, *f*, HS, R2* and T1 maps, for both region of interest and voxel-wise analysis. Color of the plot indicates whether the measurement uncertainty is dependent on both tissue type and anatomical zone (blue), tissue type only (orange), anatomical zone only (green), or independent of tissue type and anatomical zone (red)

The repeatability coefficients of all 413 radiomic features were also assessed for dependence on tissue type (tumor vs benign tissue) and anatomical zone (PZ vs nPZ) to determine the need to establish region-specific thresholds to detect true change. Figure 3 summarizes and classifies the dependence of each radiomics feature into four categories—no dependence, dependence on tissue type or anatomical region, or both. The repeatability of 38 and 61 (out of all 413 features) radiomic features measured with the ROI-based and voxel-wise approach, respectively, was found to be significantly different between benign and tumor tissues (*p* < 0.05). The repeatability of 92 and 118 radiomic features measured with ROI-based and voxel-wise approach, respectively, was found to be significantly different between the anatomical zones (*p* < 0.05). The majority of the radiomic features with dependence on tissue type or anatomical region are derived from ADC, *D*, *f*, HS, and R2* maps. Lookup tables of %RC of radiomic features derived from each qMRI parameter according to dependence of repeatability on tissue type and anatomical region are given in Supplementary Data 3.

**Fig. 4:**
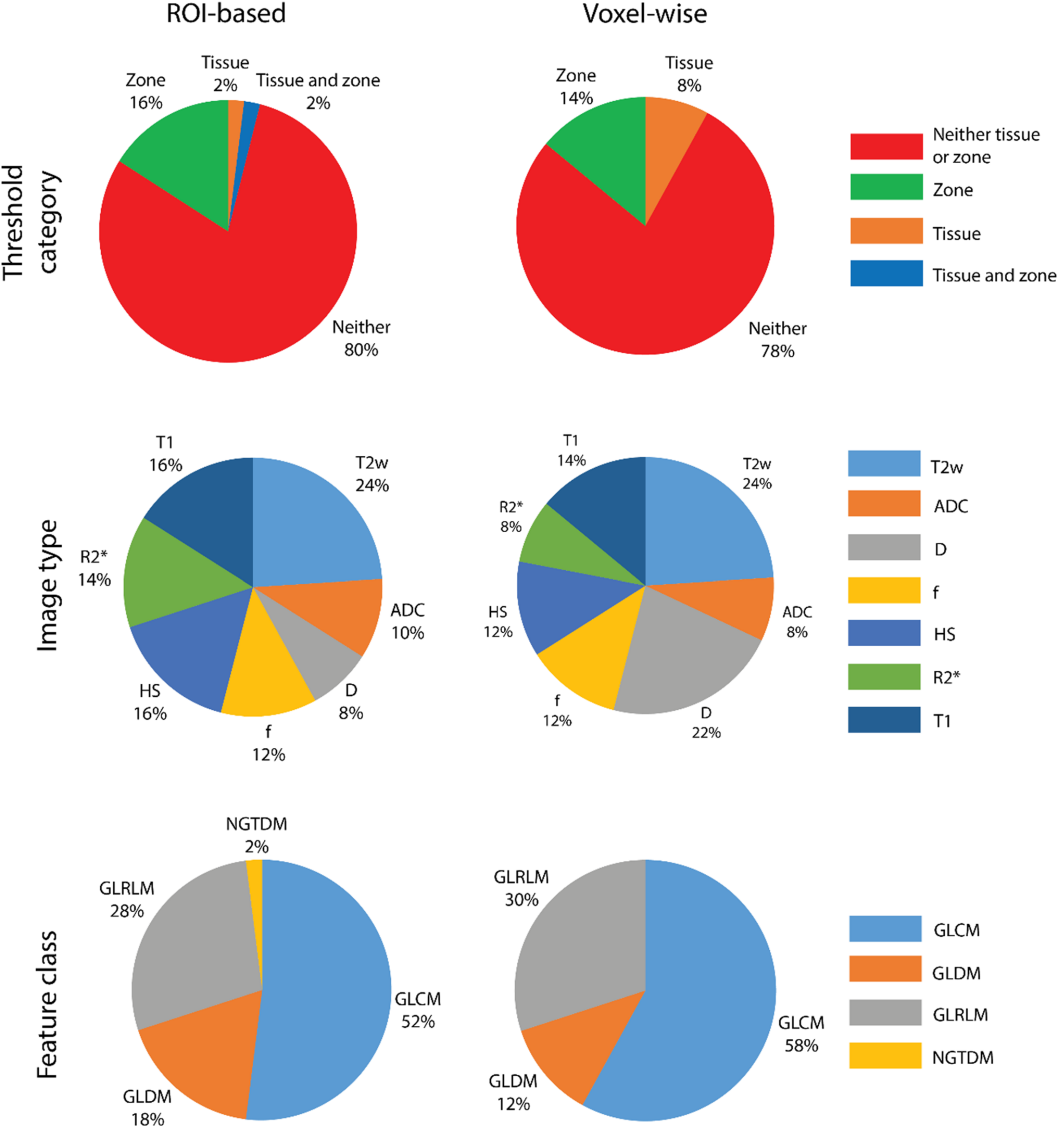
Characteristics of the top 50 features ranked by repeatability measured using the region of interest (ROI) (left column) and voxel-wise (right column) analysis. The top row indicates the percentage of features with repeatability coefficient (%RC) dependent on both tissue and zone, tissue only, anatomical zone only, or independent of tissue and anatomical zone (neither). The middle row indicates the percentage of features extracted from ADC, D, f, HS, R2*, T1 maps and T2w images. The bottom row indicates the percentage of features from the gray level co-occurrence matrix (GLCM), gray level run length matrix (GLRLM, gray level dependence matrix (GLDM), and the neighboring gray tone difference matrix (NGTDM) classes

Figure 4 shows the characteristics of the 50 radiomic features with the smallest %RCs, selected separately for the ROI and voxel-wise measurement approaches, with the values summarized in Supplementary Data 4. The %RC of the top 50 features ranged from 0.02 to 7.4% when measured using the ROI-based approach, and 0.3–27% when measured voxel-wise. For nearly 80% of the features, repeatability was not dependent on either the tissue type or the anatomical region. Thirty-six features of the top 50 were found in common between the ROI and voxel-wise measurement approaches. The repeatability of 24 of the 36 common top features between the measurement approaches was not dependent on tissue type, or the anatomical region. In the remaining 12 of 36 common features, 4 features had the same dependency on tissue type or anatomical region between the ROI and voxel-wise measurement approaches. The largest number of features in the top 50 most repeatable features was derived from T2w images. All qMRI parameter maps contributed to the top 50 most repeatable features, including inherently noisy parametric maps, such as R2* and *f*. The GLCM feature class contributed to approximately half of the top 50 most repeatable features, followed by the GLRLM, GLDM and NGTDM feature classes.

## Discussion

In this work, we conducted a comprehensive repeatability analysis and reported %RC values for several qMRI parameters and radiomic features in the prostate for both ROI and voxel-wise analysis approaches. Our results indicate that %RC estimates can vary significantly in both tissue type (tumor vs benign tissue) and anatomical zone (PZ vs nPZ) in several qMRI parameters and radiomic features. While the values of %RC may not be directly applicable to all patient cohorts (our cohorts included intermediate risk patients and healthy volunteers) and image acquisition protocols, our work demonstrates the magnitude of %RC will be variable in tissue type and anatomical zone. In this study, we have provided a framework for determining the threshold for minimum detectable change in qMRI parameters and radiomic features in longitudinal studies, according to their differences between anatomical zone (PZ vs. nPZ) and tissue type (benign vs tumor). Furthermore, we discuss below, how these values may vary under different circumstances.

In this study, we showed that both ROI and voxel-wise measurements of ADC and *D* in the benign tissue regions were significantly more repeatable (lower %RC) compared to those obtained in tumor tissue regions. This is similar to findings from previous studies that observed %RC of ADC in tumors to be up to 1.5 times higher than in normal prostate tissue [26, 30]. This means that when using DWI for clinical interpretation, for the same difference in ADC measured in both tissues, it will be more challenging to differentiate treatment-related effects from measurement uncertainties in tumor regions. This is likely due to the smaller size of tumors (< 5 cm^3^ in the test–retest patient cohort) compared to benign tissue. Previous studies have reported an inverse correlation between %RC and ROI size and a rapid increase of %RC below an ROI of 2 cm^3^ in DWI-derived qMRI parameters [46, 60]. Given the small sample size (*n* = 10) in this study, the dependence of %RC on tumor size could not be further evaluated. However, this dependence may be a consequence of imperfect co-registration of test–retest images, which can have a larger detrimental effect on smaller regions of interest [61]. Furthermore, the effect of the mismatch of smaller regions is only significant in the case of ADC and *D*, whose absolute values are also significantly different between tumor and benign tissues unlike the remaining qMRI parameters (Supplementary Data 1). The mode of analysis (ROI vs voxel-wise) resulted only in a significant increase in %RC of these parameters but did not affect their dependence on tissue type. Interestingly, a recent study by Fernando et al. reported that, using a 1.5 T MRI linear accelerator, the voxel-wise measurement of ADC was more repeatable in the tumor compared to the benign PZ [62]. This discrepancy may be attributed to two factors. First, their study did not specifically compare %RC between tissue types, and hence did not separate tumors by anatomical zone. Second, unlike this study, their study did not erode the whole-gland segmentation, potentially leading to the PZ %RC influenced by non-prostate tissue variability. Nevertheless, these findings indicate that %RCs should be established separately for tumor and benign tissues for qMRI parameters ADC and *D*.

Repeatability coefficients of ADC, *D*, and HS measurements using the ROI approach were found to be dependent on the anatomical zone and were consistently higher in the PZ. The dependence on anatomical zone potentially results from the proximity of PZ to the rectum. Rectal gas leads to severe image distortion and signal loss due to the tissue-air susceptibility differences [45, 47, 63], which is more likely to manifest in the PZ. Given the transient nature of rectal gas, repeatability of DWI-derived qMRI parameter measurements in the PZ is lower than in the nPZ. The artifacts could be controlled by the use of imaging sequences less prone to susceptibility and motion artifacts, such as non-EPI-based DWI, enema procedures prior to the imaging scan, and an endorectal coil during image acquisition [64–67]. However, in consideration of patient comfort in this longitudinal study with repeated MRI scans, enemas and endorectal coils were not used. As the DWI in this study was performed using echo-planar readouts [68–70], these images were the most affected by the artifacts in the PZ, consequently resulting in a two-fold increase in %RC of qMRI parameter ROI measurements in the PZ compared to the nPZ. However, the repeatability of voxel-wise analysis of ADC, *D*, and HS was not dependent on the anatomical zones. The reason for this is unclear as factors contributing to uncertainty in qMRI parameters such as artifacts due to air–tissue susceptibility mismatch and errors in registration to compensate anatomical deformations are expected to affect both modes of analysis. Yet, our results suggest that for voxel-wise analysis, the %RC is comparable between PZ and nPZ, and establishing a repeatability threshold for each tissue type (tumor and benign) in the entire prostate gland is sufficient for qMRI parameters derived from DWI. Similarly, qMRI parameters R2* and *f* showed comparable repeatability between anatomical zones in both modes of analysis despite also being derived from imaging sequences prone to susceptibility and motion artifacts. This may be attributed to the lower overall repeatability of R2* and *f* relative to other qMRI parameters, reducing the impact of anatomical zone-specific uncertainties.

Repeatability of radiomic features is known to depend on several factors, such as the type of image or qMRI parameter map [35, 71], image acquisition parameter including echo time, acceleration and voxel size [72–75], and processing of the image including normalization, types of filters, and the registration method [35, 76, 77]. Several studies have reported the repeatability of radiomic features derived from T2w and ADC in PCa [32, 35–37]. However, repeatability of these features in benign tissue is largely unknown. In this study, we reported the measured uncertainty values in benign tissues and found that %RCs of 38 and 61 (out of the entire set of 413) radiomic features, evaluated using an ROI-based and voxel-wise approach, respectively, were significantly different from the tumor tissues. These features were predominantly derived from ADC, *D* and HS parametric maps and were potentially impacted by the differing repeatability of these parameters among the tissue types. At the voxel-wise level of measurements, we found that 20 radiomic features derived from R2* maps showed significantly different repeatability between tumor and benign tissue. However, the cause remains unclear as the R2* measurements and its repeatability did not differ significantly between tissue types. In this study, the %RC of 92 and 118 features (out of all 413) from ROI and voxel-wise analysis, respectively, was found to be significantly different between the anatomical zones. The dominant source of these features was from ADC, *f*, and HS in both modes of analysis, and R2* in voxel-wise analysis. ADC and *D* are often assumed to reflect diffusion. However, there are differences in the *b* values used in the estimation of ADC (50 and 800 s/mm^2^) and *D* (200, 300, 400, 600, 800 s/mm^2^). The *b* values for ADC estimation in our study were chosen due to the common use in clinical practice, while *D* was calculated using higher *b* values to account for the pseudo-diffusion effect at low *b* values. Hence, we acknowledge that our ADC values are likely to be influenced by the perfusion effect that is more likely to be present in the DWI signal at *b* < 200 s/mm^2^. As our results show, this difference is also observed in the repeatability of radiomic features, with those derived ADC and *f,* having near identical anatomical zone dependence in repeatability, and those derived from *D* maps showing no significant dependence on the anatomical zone. It should be noted that the dependency of the %RC on tissue type or anatomical zone does not account for the repeatability of the features, which is further discussed below.

In this study, we presented the top 50 most repeatable features (with the smallest %RCs) of ADC, *D*, *f*, HS, R2*, T1 maps and T2w images as potential QIBs of treatment response. In contrast to previous studies, the GLCM feature class was found to contribute to nearly half of the top 50 most repeatable features. While the cause of this is unclear, it has been previously reported that the choice of evaluation metric (RC vs intra-class or concordance correlation coefficients) [77] can result in difference in radiomic features identified as robust. Interestingly, for the majority of the top 50 features, the repeatability was independent of both tissue type and anatomical region, even for features extracted from qMRI parameters whose %RC values were found to depend on tissue type and anatomical zone. This implies that factors responsible for the regional variation in repeatability, such as image artifacts and imperfection in registration, could also decrease the reliability of the radiomic features. While radiomics analysis can potentially extract robust features that are less prone to uncertainty arising from artifacts and noise in the original image/parametric map, low %RC values do not necessarily imply the potential to detect treatment response. Identification of candidate radiomic features for predicting treatment response therefore requires interpretation of the results of this study in conjunction with delta-radiomics data in future longitudinal imaging studies.

There are a few limitations in this study. First, imaging was performed on scanners from a single vendor, and a single magnetic field strength, and a single %RC was established in this multi-scanner study. As repeatability of qMRI is known to depend on image acquisition parameters as well as scanner hardware, the use of a single, study-specific %RC may not be generalizable to multi-vendor, multi-field strength studies. Ideally, the repeatability of imaging biomarkers should be standardized to enable universal translation into clinical practice. In practice, however, this is challenging due to variations in hardware and sequence implementation between scanners and vendors. Future multi-scanner longitudinal imaging studies should therefore characterize the %RC for each scanner independently to determine whether a single, study-specific %RC can be applied across all datasets. Addition of more sites and scanners in future studies to further characterize and establish %RCs can enable the translation of qMRI-based imaging biomarkers to a wider variety of scanners.

A second limitation of this study is that only rigid registration was used to correct for motion between each of the imaging sequences at each scanning session. While deformable image registration was used to register the longitudinal scans to the baseline scan reference space, this was only applied for co-registration of the 3D sagittal T2w image obtained at each imaging session. Sources of non-rigid movement and deformation of the prostate, such as bladder filling, rectal motion and gas movement [78, 79], and distortions to the prostate caused by rectal gas susceptibility artifact [70], cannot be corrected with rigid registration. Furthermore, no motion correction was applied between the *b* values in DWI, echo times in R2* mapping, and flip angles in T1 mapping. Methods to retrospectively correct for non-rigid deformation of the prostate can include the use of B0 mapping or reverse gradient polarity techniques for distortion correction in DWI. However, these methods require acquisition of additional images during the scanning session. Alternatively, deformable image registration can be used for correction of distortion and motion. While the feasibility of deformably registering DWI to T2w images has been demonstrated in the prostate [80, 81], the impact of these processes on the image signal intensity, and hence the accuracy of the derived qMRI parameters and radiomic features, is unclear and should be investigated in future work.

Third, although highly significant differences in repeatability of radiomic features were found between anatomical zones and tissue types, the statistical power of these results is limited by the small sample size compared to the number of features. Although the number of subjects is comparable with previous studies (*n* < 20 for majority of studies [26, 30, 33, 35, 58, 60, 82–85]), future investigations with larger numbers of subjects are required to confirm the results regarding the dependence of radiomics feature %RC on anatomical zone and tissue type.

Lastly, the %RCs in this study were estimated from treatment naïve subjects and may not be representative of the true uncertainties in irradiated prostates. Changes in qMRI parameters and radiomic features during or after RT with similar magnitudes as the %RCs established based on treatment naïve subjects should therefore be interpreted with caution.

## Conclusion

This is the first study to establish %RCs for both ROI and voxel-wise measurements of an extensive range of qMRI parameters and radiomic features derived from T2w images, DWI, R2* and T1 maps based on differences in repeatability between tissue types (tumor vs benign tissue) and the anatomical regions (PZ vs. nPZ). DWI-derived parameters require twice the level of change in the tumor tissue and the PZ to indicate a true difference, compared to benign tissue and nPZ, respectively. In contrast, the repeatability of T1 maps and T2w image-based features remains unaffected by tissue types and zones. At the voxel level, ADC and *D* again showed higher %RC in tumor regions compared to benign tissue, meaning a higher threshold should be applied to interpret treatment-related changes. Parameters and features with comparable %RC between tissue types and anatomical zones, such as T1 maps and their radiomic features, could have one uncertainty threshold established for the entire prostate gland. Further research is required to validate the reliability of the parameters and features to differentiate treatment effects from measurement uncertainties and enable clinical translation of these QIBs for real-time adaptive RT and longitudinal response monitoring.

## Supplementary Information

Below is the link to the electronic supplementary material.

Supplementary file1 (PDF 876 KB)

## Data Availability

The tabulated qMRI parameter and the radiomic feature measurements that support the findings of this study are available from the authors on reasonable request.
