# Supplementary material for: Anatomical zone and tissue type impacts the repeatability of quantitative MRI parameters and radiomic features for longitudinal monitoring of treatment response in the prostate
Source: MAGMA. 2025 Feb 22;38(3):475–90. doi: 10.1007/s10334-025-01231-9 (PMC12255548; doi:10.1007/s10334-025-01231-9)
Supplement: Supplementary file 1 — Supplementary file1 (PDF 876 KB) [file 10334_2025_1231_MOESM1_ESM.pdf]

Table of Contents

Supplementary Methods 3

Image registration methods 3

Supplementary Figures 4

Supplementary Figure 1 Example of registered test-retest T2-weighted images, apparent diffusion coefficient, diffusion coefficient, perfusion fraction, hypoxia score, transverse relaxation rate, and longitudinal relaxation time maps of a volunteer. 4

Supplementary Data 1 5

Table S1-1 Mean [95% confidence interval] of qMRI parameters in the volunteer and patient cohorts in region of interest (ROI) measurements within the anatomical regions (peripheral zone, PZ, and non-peripheral zone, nPZ) and between tissue type (patient only, tumor and benign). 5

Supplementary Data 2 6

Table S2-1 Repeatability coefficients (%RC) for mean region of interest (ROI) measurement of qMRI parameters in the whole prostate gland (WG), peripheral zone (PZ) and non-peripheral zone (nPZ). Significant differences in %CV between anatomical regions (peripheral zone, PZ, and non-peripheral zone, nPZ) in the combined volunteer and patient data, and between tissue type (tumor and benign) in the patient data are shown in bold. 6

Table S2-2 Repeatability coefficients (%RC) of voxel-wise quantitative parameter values. Significant differences between peripheral zone and non-peripheral zone are shown in bold. Significant differences in %wCV (obtained per participant) between anatomical regions (peripheral zone, PZ, and non-peripheral zone, nPZ) in the combined volunteer and patient data, and between tissue type (tumor and benign) in the patient data are shown in bold. 7

Supplementary Data 3 9

Table S3-3 Repeatability coefficients (%RC) of region of interest (ROI) and voxel-wise measurements of radiomic features derived from T2w images stratified by their dependency to tissue type (benign and tumor) and anatomical zone (peripheral zone, PZ, and non-peripheral zone, nPZ). 9

Table S3-4 Repeatability coefficients (%RC) of region of interest (ROI) and voxel-wise measurements of radiomic features derived from ADC map stratified by their dependency to tissue type (benign, B, and tumor, T) and anatomical zone (peripheral zone, PZ, and non-peripheral zone, nPZ). 12

Table S3-5 Repeatability coefficients (%RC) of region of interest (ROI) and voxel-wise measurements of radiomic features derived from D map stratified by their dependency to tissue type (benign, B, and tumor, T) and anatomical zone (peripheral zone, PZ, and non-peripheral zone, nPZ)... 15

Table S3-6 Repeatability coefficients (%RC) of region of interest (ROI) and voxel-wise measurements of radiomic features derived from f map stratified by their dependency to tissue type (benign, B, and tumor, T) and anatomical zone (peripheral zone, PZ, and non-peripheral zone, nPZ)... 18

Table S3-7 Repeatability coefficients (%RC) of region of interest (ROI) and voxel-wise measurements of radiomic features derived from HS map stratified by their dependency to tissue type (benign, B, and tumor, T) and anatomical zone (peripheral zone, PZ, and non-peripheral zone, nPZ)... 21

Table S3-8 Repeatability coefficients (%RC) of region of interest (ROI) and voxel-wise measurements of radiomic features derived from R2* map stratified by their dependency to tissue type (benign, B, and tumor, T) and anatomical zone (peripheral zone, PZ, and non-peripheral zone, nPZ)... 24

Table S3-9 Repeatability coefficients (%RC) of region of interest (ROI) and voxel-wise measurements of radiomic features derived from T1 map stratified by their dependency to tissue type (benign, B, and tumor, T) and anatomical zone (peripheral zone, PZ, and non-peripheral zone, nPZ)... 27

Supplementary Data 4 30

Table S4-1 Top 50 most repeatable radiomics features measured using ROI approach. Features are sorted by the image/map it was extracted from, feature class, and feature name. The threshold category indicates whether the measurement uncertainty is dependent on both tissue and region, tissue only, anatomical region only, or independent of tissue and region (whole gland). Highlighted features indicate that the same feature was ranked in the top 50 in both ROI and voxel-wise measurement approaches. 30

Table S4- 2 Top 50 most repeatable radiomics features measured using voxel-wise approach. Features are sorted by the image/map it was extracted from, feature class, and feature name. The threshold category indicates whether the measurement uncertainty is dependent on both tissue and region, tissue only, anatomical region only, or independent of tissue and region (whole gland). Highlighted features indicate that the same feature was ranked in the top 50 in both ROI and voxel-wise measurement approaches. 32

Supplementary Methods

Image registration methods

Software used: Elastix module in 3D Slicer (5.0.3)

Rigid registration parameters

Method: MultiResolutionRegistration

Optimizer: AdaptiveStochasticGradientDescent

Transform: EulerTransform

ImagePyramidSchedule: 8 8 2 4 4 1 1 1 0.5

Cost Function: AdvancedMattesMutualInformation

Resampling interpolator: Linear

Deformable registration parameters

Method: MultiResolutionRegistration

Optimizer: AdaptiveStochasticGradientDescent

Transform: BSplineTransform

Grid spacing: 100/50/25/10 mm

Cost Function: AdvancedMattesMutualInformation

Resampling interpolator: Linear

Supplementary Figures

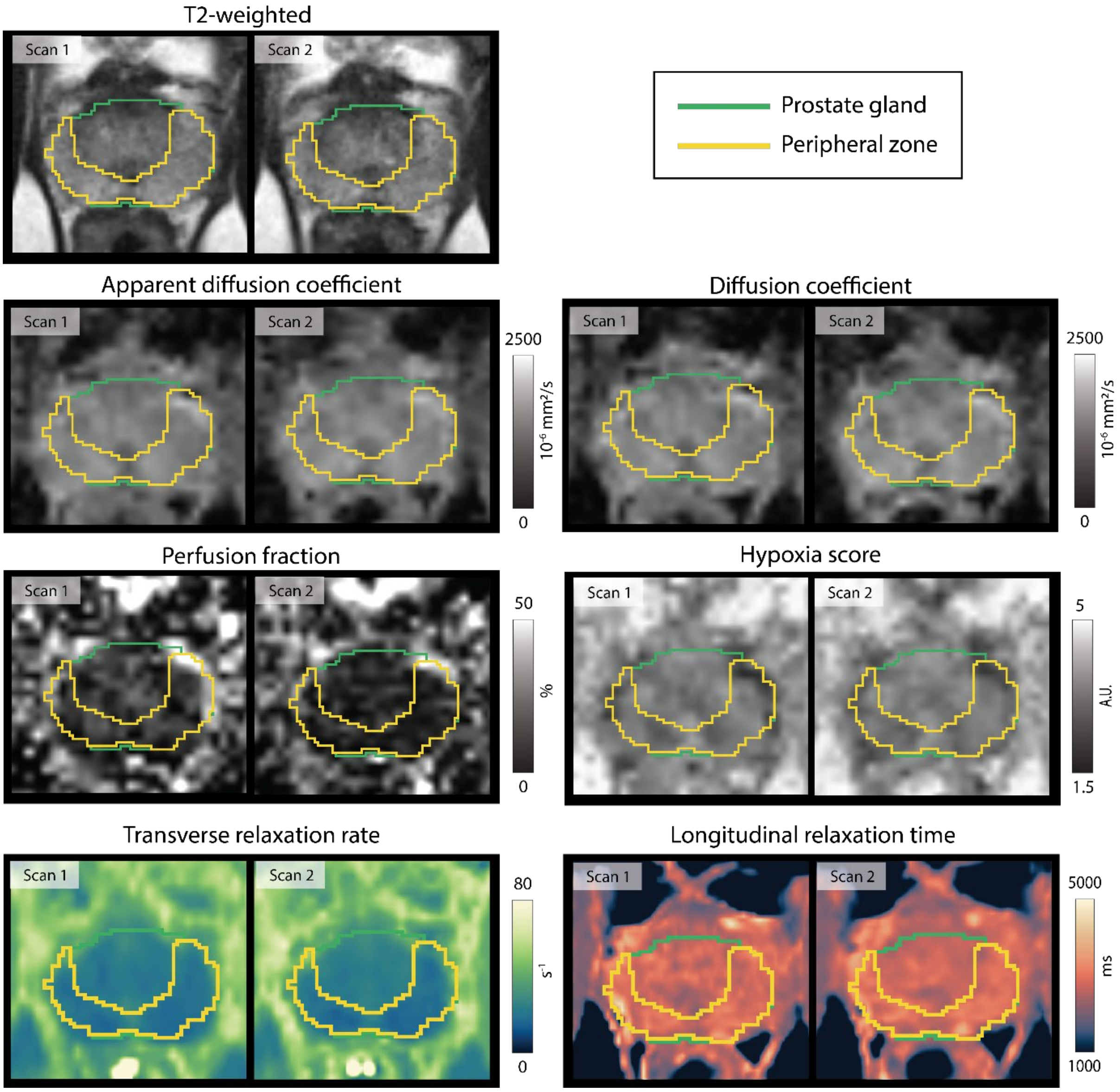

Supplementary Figure 1 Example of registered test-retest T2-weighted images, apparent diffusion coefficient, diffusion coefficient, perfusion fraction, hypoxia score, transverse relaxation rate, and longitudinal relaxation time maps of a volunteer. All images were registered to the sagittal T2-weighted image of the first scan. The prostate gland and peripheral zone, delineated on the sagittal T2-weighted image of the first scan, were propagated to each qMRI parameter map and axial T2-weighted image.

Supplementary Data 1

Table S1-1 Mean [95% confidence interval] of qMRI parameters in the volunteer and patient cohorts in region of interest (ROI) measurements within the anatomical regions (peripheral zone, PZ, and non-peripheral zone, nPZ) and between tissue type (patient only, tumor and benign).

	ADC (10^{-6} mm^2/s)	*D* (10^{-6} mm^2/s)	*f* (%)	*HS* (arbitrary units)	*R2** (s^{-1})	*T1* (ms)
Volunteers						
WG	1138 [1075 - 1201]	1182 [1143 - 1221]	13.0 [12.1 - 13.9]	3.2 [3.1 - 3.2]	32.0 [26.7 - 37.3]	2669 [2616 - 2722]
PZ	1190 [1123 - 1256]	1234 [1177 - 1292]	12.9 [11.8 - 14.0]	3.1 [3.0 - 3.2]	32.6 [25.9 - 39.4]	2784 [2720 - 2847]
nPZ	1094 [1036 - 1153]	1144 [1113 - 1174]	13.2 [12.2 - 14.2]	3.2 [3.2 - 3.3]	31.6 [27.6 - 35.7]	2578 [2530 - 2627]
Patients						
WG						
Benign	1353.0 [1311.6 - 1394.3]	1371.0 [1328.8 - 1413.3]	10.9 [10.2 - 11.6]	3.0 [2.9 - 3.0]	26.6 [24.2 - 29.0]	2474.6 [2331.6 - 2617.7]
Tumor	1113.9 [1061.3 - 1166.5]	1138.0 [1085.5 - 1190.6]	10.5 [9.6 - 11.5]]	3.3 [3.2 - 3.4]	27.9 [25.8 - 30.0]	2512.8 [2423.8 - 2601.7]
PZ						
Benign	1415.4 [1358.3 - 1472.5]	1440.0 [1376.2 - 1503.9]	10.5 [9.6 - 11.5]	2.9 [2.8 - 3.0]	29.2 [25.6 - 32.8]	2108.8 [1707.0 - 2510.7]
tumor	1233.2 [1163.2 - 1303.1]	1269.7 [1202.4 - 1337.0]	10.2 [8.8 - 11.7]	3.1 [3.0 - 3.2]	32.7 [27.4 - 38.0]	2254.2 [1955.4 - 2553.0]
nPZ						
Benign	1317.8 [1280.8 - 1354.7]	1335.3 [1300.4 - 1370.2]	11.0 [10.2 - 11.8]	3.0 [3.0 - 3.1]	25.5 [23.6 - 27.4]	2564.4 [2496.2 - 2632.7]
Tumor	1088.6 [1028.7 - 1148.5]	1110.3 [1048.9 - 1171.6]	10.6 [9.7 - 11.6]	3.3 [3.3 - 3.4]	27.1 [25.4 - 28.8]	2553.2 [2479.1 - 2627.2]

WG – whole gland, PZ – peripheral zone, nPZ – non-peripheral zone

Supplementary Data 2

Table S2-1 Repeatability coefficients (%RC) for mean region of interest (ROI) measurement of qMRI parameters in the whole prostate gland (WG), peripheral zone (PZ) and non-peripheral zone (nPZ). Significant differences in %CV between anatomical regions (peripheral zone, PZ, and non-peripheral zone, nPZ) in the combined volunteer and patient data, and between tissue type (tumor and benign) in the patient data are shown in bold.

	ADC (10^{-6} mm^2/s)	*D* (10^{-6} mm^2/s)	*f* (%)	*HS* (arbitrary units)	*R2** (s^{-1})	*T1* (ms)
Patients and volunteer						
WG	5.8	5.8	26.2	3.2	38.5	10.3
PZ	**13.4**	**13.5**	41.1	**7.9**	46.9	14.1
nPZ	**6.2**	**5.0**	27.9	**3.2**	35.0	9.7
Paired t-test p-value	**0.002**	**0.002**	0.19	**0.015**	0.10	0.60
Patients only						
WG						
Benign	**6.9**	7.1	24.9	4.2	28.4	20.7
Tumor	**17.3**	12.9	33.8	6.1	30.5	8.6
Paired t-test p-value	**0.03**	0.07	0.43	0.40	0.30	0.18
PZ						
Benign	15.4	16.1	39.9	8.6	34.4	15.4
Tumor	29.4	26.7	73.3	11.6	27.9	16.3
Paired t-test p-value	0.20	0.14	0.14	0.60	0.94	0.21
nPZ						
Benign	**6.4**	**6.5**	25.3	3.6	28.6	14.0
Tumor	**13.4**	**11.4**	33.4	4.4	22.9	6.9
Paired t-test p-value	**0.01**	**0.002**	0.46	0.76	0.57	0.16

WG – whole gland, PZ – peripheral zone, nPZ – non-peripheral zone

Table S2-2 Median and range of repeatability coefficients (%RC) of voxel-wise quantitative parameter values. Significant differences between peripheral zone and non-peripheral zone are shown in bold. Significant differences in %wCV (obtained per participant) between anatomical regions (peripheral zone, PZ, and non-peripheral zone, nPZ) in the combined volunteer and patient data, and between tissue type (tumor and benign) in the patient data are shown in bold.

	ADC (10^{-6} mm^2/s)	*D* (10^{-6} mm^2/s)	*f* (%)	*HS* (arbitrary units)	*R2** (s^{-1})	*T1* (ms)
WG	32.7 [17.3 – 52.7]	30.2 [16.9 – 38.1]	127.8 [94.6 – 158.1]	18.9 [10.9 – 26.6]	55.2 [27.4 – 122.0]	24.7 [16.8 – 77.4]
PZ	33.2 [17.3 – 64.8]	31.6 [16.5 – 66.0]	131.1 [95.4 – 164.9]	16.3 [10.6 – 38.9]	55.5 [30.3 – 123.5]	25.9 [16.3 – 63.4]
nPZ	31.1 [17.3 – 49.7]	25.9 [16.8 – 39.6]	126.7 [94.1 – 161.5]	15.8 [10.6 – 29.2]	49.8 [22.3 – 136.3]	22.0 [16.6 – 81.8]
Paired t-test p-value	0.07	0.10	0.77	0.26	0.89	0.18
Patients only WG Benign	**28.6 [15.8 – 36.6]**	**28.2 [16.1 – 37.4]**	129.5 [92.5 – 163.2]	18.5 [11.1 – 28.8]	50.3 [29.1 – 135.2]	27.7 [18.8 – 86.4]
Tumor	**42.8 [29.9 – 83.1]**	**37.7 [24.7 – 78.4]**	137.5 [108.3 – 194.5]	19.2 [10.5 – 44.0]	47.2 [24.7 – 140.7]	27.5 [19.4 – 50.1]
Paired t-test p-value	**0.011**	**0.023**	0.17	0.45	0.40	0.28
PZ Benign	30.5 [16.9 – 45.4]	31.2 [16.3 – 49.6]	128.7 [90.0 – 165.1]	17.2 [11.6 – 27.7]	54.2 [35.5 – 83.7]	28.0 [21.1 – 61.5]
Tumor	46.0 [25.5 – 111.0]	31.5 [20.8 – 111.9]	122.0 [106.4 – 231.3]	16.1 [10.5 – 60.4]	54.1 [30.5 – 87.3]	28.7 [19.1 – 70.1]
Paired t-test p-value	0.064	0.17	0.15	0.37	0.45	0.72
nPZ Benign	**27.3 [15.0 – 36.8]**	**24.5 [15.5 – 38.2]**	130.2 [91.4 – 169.5]	16.2 [10.8 – 31.0]	47.6 [25.5 – 150.4]	27.8 [17.5 – 90.6]
Tumor	**43.4 [26.6 – 67.6]**	**36.6 [24.9 – 59.3]**	149.3 [108.3 – 176.2]	19.4 [10.0 – 30.5]	34.5 [22.2 – 144.0]	23.8 [17.2 – 54.3]
Paired t-test p-value	**0.001**	**0.002**	0.22	0.57	0.20	0.23

WG – whole gland, PZ – peripheral zone, nPZ – non-peripheral zone

Supplementary Data 3

Table S3-3 Repeatability coefficients (%RC) of region of interest (ROI) and voxel-wise measurements of radiomic features derived from T2w images stratified by their dependency to tissue type (benign and tumor) and anatomical zone (peripheral zone, PZ, and non-peripheral zone, nPZ).

$\%RC_{ROI}$				
**tissue and zone**				
			PZ	nPZ
**ngtdm**	Coarseness	benign	10.5	7.9
		tumor	13.7	5.9
**zone**				
			PZ	nPZ
**glcm**	ClusterShade	all	319.6	79.5
**gldm**	LargeDependenceEmphasis	all	23.0	17.6
**glrlm**	RunVariance	all	30.9	21.8
**tissue**				
			Whole gland	
**glcm**	Autocorrelation	benign	34.1	
		tumor	39.9	
	Correlation	benign	29.3	
		tumor	42.4	
	JointAverage	benign	17.3	
		tumor	20.4	
	SumAverage	benign	17.3	
		tumor	20.4	
**gldm**	DependenceEntropy	benign	1.3	
		tumor	0.9	
	HighGrayLevelEmphasis	benign	33.1	

Median $\%RC_{voxel}$				
**tissue and zone**				
			PZ	nPZ
**glrlm**	GrayLevelNonUniformity	benign	356.1	355.1
		tumor	355.3	354.4
	RunLengthNonUniformity	benign	356.5	355.6
		tumor	355.8	355.0
**zone**				
			PZ	nPZ
**glrlm**	GrayLevelNonUniformityNormalized	all	41.3	36.3
**tissue**				
			Whole gland	
**glcm**	Id	benign	142.9	
		tumor	132.0	
	Idm	benign	142.9	
		tumor	132.0	
	Idn	benign	2.7	
		tumor	2.4	
	InverseVariance	benign	29.8	
		tumor	29.4	
**whole gland**				
			Whole gland	

		tumor	39.0
**glrlm**	HighGrayLevelRunEmphasis	benign	32.9
		tumor	38.8
	LongRunLowGrayLevelEmphasis	benign	39.0
		tumor	47.6
	ShortRunHighGrayLevelEmphasis	benign	32.7
		tumor	39.6

whole gland

			Whole gland
**glcm**	ClusterProminence	all	65.9
	ClusterTendency	all	31.8
	Contrast	all	37.0
	DifferenceAverage	all	19.1
	DifferenceEntropy	all	9.8
	DifferenceVariance	all	35.8
	Id	all	7.9
	Idm	all	11.1
	Idmn	all	0.2
	Idn	all	1.1
	Imc1	all	12.6
	Imc2	all	5.8
	InverseVariance	all	7.0
	JointEnergy	all	18.5
	JointEntropy	all	6.1
	MaximumProbability	all	15.9
	MCC	all	6.8
	SumEntropy	all	5.7
	SumSquares	all	33.6
**gldm**	DependenceNonUniformity	all	8.5
	DependenceNonUniformityNormalized	all	8.5

**glcm**	Autocorrelation	all	55.1
	ClusterProminence	all	169.4
	ClusterShade	all	34583.2
	ClusterTendency	all	99.3
	Contrast	all	95.9
	Correlation	all	55.1
	DifferenceAverage	all	52.3
	DifferenceEntropy	all	27.0
	DifferenceVariance	all	96.1
	Idmn	all	0.8
	Imc1	all	52.4
	Imc2	all	24.8
	JointAverage	all	27.6
	JointEnergy	all	60.1
	JointEntropy	all	21.5
	MaximumProbability	all	62.7
	MCC	all	31.3
	SumAverage	all	27.6
	SumEntropy	all	20.8
	SumSquares	all	93.6
**gldm**	DependenceEntropy	all	9.9
	DependenceNonUniformity	all	361.6
	DependenceNonUniformityNormalized	all	41.5
	DependenceVariance	all	97.9
	GrayLevelNonUniformity	all	42.8
	GrayLevelVariance	all	91.3
	HighGrayLevelEmphasis	all	53.0
	LargeDependenceEmphasis	all	72.3
	LargeDependenceHighGrayLevelEmphasis	all	83.2
	LargeDependenceLowGrayLevelEmphasis	all	95.0
	LowGrayLevelEmphasis	all	95.0

	DependenceVariance	all	17.4
	GrayLevelNonUniformity	all	13.1
	GrayLevelVariance	all	33.1
	LargeDependenceHighGrayLevelEmphasis	all	36.0
	LargeDependenceLowGrayLevelEmphasis	all	43.3
	LowGrayLevelEmphasis	all	39.2
	SmallDependenceEmphasis	all	14.5
	SmallDependenceHighGrayLevelEmphasis	all	35.3
	SmallDependenceLowGrayLevelEmphasis	all	41.4
**glrlm**	GrayLevelNonUniformity	all	8.6
	GrayLevelNonUniformityNormalized	all	12.2
	GrayLevelVariance	all	31.7
	LongRunEmphasis	all	10.0
	LongRunHighGrayLevelEmphasis	all	33.4
	LowGrayLevelRunEmphasis	all	39.3
	RunEntropy	all	3.1
	RunLengthNonUniformity	all	9.0
	RunLengthNonUniformityNormalized	all	5.7
	RunPercentage	all	3.5
	ShortRunEmphasis	all	2.9
	ShortRunLowGrayLevelEmphasis	all	39.2
**ngtdm**	Busyness	all	20.0
	Complexity	all	45.2
	Contrast	all	24.7
	Strength	all	24.1

	SmallDependenceEmphasis	all	52.4
	SmallDependenceHighGrayLevelEmphasis	all	77.0
	SmallDependenceLowGrayLevelEmphasis	all	77.5
**glrlm**	GrayLevelVariance	all	88.4
	HighGrayLevelRunEmphasis	all	52.6
	LongRunEmphasis	all	38.7
	LongRunHighGrayLevelEmphasis	all	58.5
	LongRunLowGrayLevelEmphasis	all	69.4
	LowGrayLevelRunEmphasis	all	53.8
	RunEntropy	all	12.6
	RunLengthNonUniformityNormalized	all	23.0
	RunPercentage	all	14.3
	RunVariance	all	90.3
	ShortRunEmphasis	all	12.2
	ShortRunHighGrayLevelEmphasis	all	56.7
	ShortRunLowGrayLevelEmphasis	all	54.5
**ngtdm**	Busyness	all	89.8
	Coarseness	all	38.3
	Complexity	all	115.6
	Contrast	all	79.6
	Strength	all	89.6

GLCM - grey level co-occurrence matrix; GLRLM - grey level run length matrix; GLDM - grey level dependence matrix; NGTDM - neighboring grey tone difference matrix.

Table S3-4 Repeatability coefficients (%RC) of region of interest (ROI) and voxel-wise measurements of radiomic features derived from ADC map stratified by their dependency to tissue type (benign, B, and tumor, T) and anatomical zone (peripheral zone, PZ, and non-peripheral zone, nPZ).

		**$\%RC_{ROI}$**		
**tissue and zone**				
			PZ	nPZ
**ngtdm**	Coarseness	benign	5.9	2.5
		tumor	9.0	8.1
**zone**				
			PZ	nPZ
**glcm**	ClusterProminence		150.1	122.9
	ClusterTendency		78.5	63.3
	DifferenceAverage		41.4	33.1
	Id		12.7	10.4
	Idm		15.0	12.6
	Imc1		22.5	18.5
	Imc2		16.2	12.7
	JointEnergy		47.6	38.7
	JointEntropy		23.0	17.9
	MaximumProbability		40.0	32.2
	MCC		15.5	12.2
	SumEntropy		19.1	14.8
	SumSquares		75.3	61.2
**gldm**	DependenceEntropy		4.2	3.1
	DependenceVariance		28.4	24.1
	GrayLevelNonUniformity		31.6	25.5
	GrayLevelVariance		76.3	62.1
	LargeDependenceEmphasis		41.9	33.7
**glrlm**	GrayLevelNonUniformity		14.1	12.1
	GrayLevelNonUniformityNormalized		29.7	24.0

		**Median $\%RC_{voxel}$**		
**tissue and zone**				
			PZ	nPZ
**glcm**	Autocorrelation		128.4	99.4
	Correlation	benign	82.1	90.5
		tumor	66.0	73.6
	JointAverage	benign	22.4	22.0
		tumor	44.5	28.7
	SumAverage	benign	22.4	22.0
		tumor	44.5	28.7
**gldm**	LowGrayLevelEmphasis	benign	43.9	44.9
		tumor	88.6	57.8
	SmallDependenceLowGrayLevelEmphasis	benign	103.2	94.9
		tumor	107.1	88.8
**glrlm**	LongRunLowGrayLevelEmphasis	benign	86.7	80.9
		tumor	99.6	86.9
	LowGrayLevelRunEmphasis	benign	43.9	45.2
		tumor	89.3	56.8
	ShortRunHighGrayLevelEmphasis	benign	57.7	55.3
		tumor	68.6	59.2
	ShortRunLowGrayLevelEmphasis	benign	62.1	55.1
		tumor	91.2	61.8
**zone**				
			PZ	nPZ
**glcm**	ClusterProminence		249.7	227.8
	ClusterTendency		169.7	149.5
	Contrast		135.9	119.7

	GrayLevelVariance		70.7	58.2
	LongRunEmphasis		36.2	28.5
	RunEntropy		7.1	5.6
	RunLengthNonUniformity		36.3	27.4
	RunLengthNonUniformityNormalized		21.6	16.7
	RunPercentage		15.1	11.5
	RunVariance		43.1	35.1
	ShortRunEmphasis		15.3	11.5
**ngtdm**	Complexity		93.6	78.0
	Strength		74.2	58.3
**tissue**				
			Whole gland	
**glcm**	Autocorrelation	benign	21.6	
		tumor	33.9	
	JointAverage	benign	10.8	
		tumor	17.3	
	SumAverage	benign	10.8	
		tumor	17.3	
**gldm**	HighGrayLevelEmphasis	benign	21.7	
		tumor	32.8	
	LargeDependenceHighGrayLevelEmphasis	benign	17.3	
		tumor	50.6	
	LowGrayLevelEmphasis	benign	20.6	
		tumor	35.7	
	SmallDependenceLowGrayLevelEmphasis	benign	23.1	
		tumor	47.6	
**glrlm**	HighGrayLevelRunEmphasis	benign	21.5	
		tumor	32.6	
	LongRunHighGrayLevelEmphasis	benign	15.2	
		tumor	40.8	

	DifferenceAverage		89.6	80.6
	DifferenceEntropy		51.7	45.3
	DifferenceVariance		109.7	94.3
	Imc1		84.5	77.9
	JointEnergy		110.8	104.8
	MaximumProbability		102.0	95.3
	SumSquares		161.0	139.7
**gldm**	GrayLevelNonUniformity		77.2	69.3
	GrayLevelVariance		161.2	138.5
	LargeDependenceLowGrayLevelEmphasis		124.7	104.7
	SmallDependenceEmphasis		95.3	87.7
	SmallDependenceHighGrayLevelEmphasis		118.8	103.5
**glrlm**	GrayLevelNonUniformityNormalized		69.5	61.6
	GrayLevelVariance		153.1	129.1
	LongRunEmphasis		81.1	75.7
	RunEntropy		21.4	19.0
	RunLengthNonUniformityNormalized		44.6	43.2
**ngtdm**	Complexity		178.4	161.3
	Strength		165.7	143.9
**tissue**				
			While gland	
**glcm**	Id	benign	75.9	
		tumor	84.5	
	Idm	benign	75.9	
		tumor	84.5	
**gldm**	HighGrayLevelEmphasis	benign	43.2	
		tumor	64.7	
	LargeDependenceHighGrayLevelEmphasis	benign	94.3	
		tumor	100.6	
**glrlm**	GrayLevelNonUniformity	benign	345.7	

	LowGrayLevelRunEmphasis	benign	20.5
		tumor	35.3
	ShortRunLowGrayLevelEmphasis	benign	16.4
		tumor	35.1
**whole gland**			
			Whole gland
**glcm**	ClusterShade		453.4
	Contrast		57.1
	Correlation		9.8
	DifferenceEntropy		19.9
	DifferenceVariance		41.7
	Idmn		0.1
	Idn		1.0
	InverseVariance		7.4
**gldm**	DependenceNonUniformity		11.4
	DependenceNonUniformityNormalized		11.4
	LargeDependenceLowGrayLevelEmphasis		65.4
	SmallDependenceEmphasis		40.6
	SmallDependenceHighGrayLevelEmphasis		71.4
**glrlm**	LongRunLowGrayLevelEmphasis		60.8
	ShortRunHighGrayLevelEmphasis		49.4
**ngtdm**	Busyness		42.7
	Contrast		24.6

		tumor	348.8
	HighGrayLevelRunEmphasis	benign	42.7
		tumor	64.1
	RunLengthNonUniformity	benign	347.7
		tumor	350.6
**whole gland**			
			Whole gland
**glcm**	ClusterShade		49154.3
	Idmn		0.4
	Idn		2.9
	Imc2		52.5
	InverseVariance		49.8
	JointEntropy		53.7
	MCC		56.2
	SumEntropy		50.1
**gldm**	DependenceEntropy		14.8
	DependenceNonUniformity		361.6
	DependenceNonUniformityNormalized		51.5
	DependenceVariance		110.9
	LargeDependenceEmphasis		96.6
**glrlm**	LongRunHighGrayLevelEmphasis		86.0
	RunPercentage		32.5
	RunVariance		108.4
	ShortRunEmphasis		32.5
**ngtdm**	Busyness		146.5
	Coarseness		59.3
	Contrast		91.8

GLCM - grey level co-occurrence matrix; GLRLM - grey level run length matrix; GLDM - grey level dependence matrix; NGTDM - neighboring grey tone difference matrix.

Table S3-5 Repeatability coefficients (%RC) of region of interest (ROI) and voxel-wise measurements of radiomic features derived from D map stratified by their dependency to tissue type (benign, B, and tumor, T) and anatomical zone (peripheral zone, PZ, and non-peripheral zone, nPZ).

			%RC$_{ROI}$
**tissue**			
			Whole gland
**gldm**	DependenceNonUniformity	benign	11.0
		tumor	16.3
	DependenceNonUniformityNormalized	benign	11.0
		tumor	16.3
	DependenceVariance	benign	22.9
		tumor	29.5
	SmallDependenceHighGrayLevelEmphasis	benign	60.2
		tumor	58.5
	SmallDependenceLowGrayLevelEmphasis	benign	27.2
		tumor	56.2
**ngtdm**	Busyness	benign	39.8
		tumor	49.2
	Coarseness	benign	3.7
		tumor	8.2
	Contrast	benign	24.0
		tumor	28.6
**whole gland**			
			Whole gland
**glcm**	Autocorrelation		36.0
	ClusterProminence		143.1
	ClusterShade		2359.0
	ClusterTendency		75.1
	Contrast		62.8
	Correlation		11.2

			Median %RC$_{voxel}$	
**tissue and zone**				
			PZ	nPZ
**glrlm**	GrayLevelNonUniformity		711.4	709.5
	RunLengthNonUniformity	benign	356.5	355.6
		tumor	355.8	355.0
**glrlm**	GrayLevelNonUniformityNormalized	all	41.3	36.3
**tissue**				
			Whole gland	
**glcm**	Id	benign	142.9	
		tumor	132.0	
	Idm	benign	142.9	
		tumor	132.0	
	Idn	benign	2.7	
		tumor	2.4	
	InverseVariance	benign	29.8	
		tumor	29.4	
**whole gland**				
			Whole gland	
**glcm**	Autocorrelation		55.1	
	ClusterProminence	all	169.4	
	ClusterShade	all	34583.2	
	ClusterTendency	all	99.3	
	Contrast	all	95.9	
	Correlation	all	55.1	
	DifferenceAverage	all	52.3	

	DifferenceAverage	38.6
	DifferenceEntropy	21.9
	DifferenceVariance	45.1
	Id	12.0
	Idm	14.3
	Idmn	0.1
	Idn	1.1
	Imc1	21.8
	Imc2	14.9
	InverseVariance	7.5
	JointAverage	17.8
	JointEnergy	44.8
	JointEntropy	21.3
	MaximumProbability	37.4
	MCC	14.3
	SumAverage	17.8
	SumEntropy	17.7
	SumSquares	71.7
**gldm**	DependenceEntropy	3.9
	GrayLevelNonUniformity	29.7
	GrayLevelVariance	72.7
	HighGrayLevelEmphasis	35.9
	LargeDependenceEmphasis	39.1
	LargeDependenceHighGrayLevelEmphasis	31.0
	LargeDependenceLowGrayLevelEmphasis	61.9
	LowGrayLevelEmphasis	34.0
	SmallDependenceEmphasis	44.7
**glrlm**	GrayLevelNonUniformity	13.9
	GrayLevelNonUniformityNormalized	28.0
	GrayLevelVariance	67.8
	HighGrayLevelRunEmphasis	35.8

	DifferenceEntropy	all	27.0
	DifferenceVariance	all	96.1
	Idmn	all	0.8
	Imc1	all	52.4
	Imc2	all	24.8
	JointAverage	all	27.6
	JointEnergy	all	60.1
	JointEntropy	all	21.5
	MaximumProbability	all	62.7
	MCC	all	31.3
	SumAverage	all	27.6
	SumEntropy	all	20.8
	SumSquares	all	93.6
**gldm**	DependenceEntropy	all	9.9
	DependenceNonUniformity	all	361.6
	DependenceNonUniformityNormalized	all	41.5
	DependenceVariance	all	97.9
	GrayLevelNonUniformity		42.8
	GrayLevelVariance	all	91.3
	HighGrayLevelEmphasis	all	53.0
	LargeDependenceEmphasis	all	72.3
	LargeDependenceHighGrayLevelEmphasis	all	83.2
	LargeDependenceLowGrayLevelEmphasis	all	95.0
	LowGrayLevelEmphasis	all	95.0
	SmallDependenceEmphasis	all	52.4
	SmallDependenceHighGrayLevelEmphasis	all	77.0
	SmallDependenceLowGrayLevelEmphasis	all	77.5
**glrlm**	GrayLevelVariance	all	88.4
	HighGrayLevelRunEmphasis	all	52.6
	LongRunEmphasis	all	38.7
	LongRunHighGrayLevelEmphasis	all	58.5

	LongRunEmphasis	33.7
	LongRunHighGrayLevelEmphasis	25.9
	LongRunLowGrayLevelEmphasis	56.5
	LowGrayLevelRunEmphasis	33.8
	RunEntropy	6.7
	RunLengthNonUniformity	32.9
	RunLengthNonUniformityNormalized	19.9
	RunPercentage	13.9
	RunVariance	41.4
	ShortRunEmphasis	13.7
	ShortRunHighGrayLevelEmphasis	44.5
	ShortRunLowGrayLevelEmphasis	28.2
**ngtdm**	Complexity	88.8
	Strength	70.5

	LongRunLowGrayLevelEmphasis	all	69.4
	LowGrayLevelRunEmphasis	all	53.8
	RunEntropy	all	12.6
	RunLengthNonUniformityNormalized	all	23.0
	RunPercentage	all	14.3
	RunVariance	all	90.3
	ShortRunEmphasis	all	12.2
	ShortRunHighGrayLevelEmphasis	all	56.7
	ShortRunLowGrayLevelEmphasis	all	54.5
**ngtdm**	Busyness	all	89.8
	Coarseness	all	38.3
	Complexity	all	115.6
	Contrast	all	79.6
	Strength	all	89.6

GLCM - grey level co-occurrence matrix; GLRLM - grey level run length matrix; GLDM - grey level dependence matrix; NGTDM - neighboring grey tone difference matrix.

Table S3-6 Repeatability coefficients (%RC) of region of interest (ROI) and voxel-wise measurements of radiomic features derived from f map stratified by their dependency to tissue type (benign, B, and tumor, T) and anatomical zone (peripheral zone, PZ, and non-peripheral zone, nPZ).

	**$\%RC_{ROI}$**			
**tissue and zone**				
			PZ	nPZ
**glcm**	Imc1	benign	24.3	11.5
		tumor	32.5	18.7
**gldm**	DependenceEntropy	benign	2.7	1.4
		tumor	5.0	2.3
**zone**				
			PZ	nPZ
**glcm**	ClusterProminence		136.5	88.5
	ClusterTendency		71.8	44.0
	Contrast		63.7	41.2
	Correlation		9.7	5.4
	DifferenceAverage		34.6	21.2
	DifferenceEntropy		17.5	10.8
	DifferenceVariance		55.2	38.6
	Id		14.2	9.0
	Idm		19.8	12.8
	Idmn		0.4	0.3
	Idn		1.8	1.3
	Imc2		8.6	3.9
	JointEnergy		34.7	19.5
	JointEntropy		11.8	6.1
	MaximumProbability		30.8	17.9
	MCC		11.1	6.0
	SumEntropy		11.2	6.0

	**Median $\%RC_{voxel}$**			
**tissue and zone**				
			PZ	nPZ
**glcm**	ClusterShade	benign	21835.6	59803.6
		tumor	3433.9	19633.0
**zone**				
			PZ	nPZ
**glcm**	ClusterProminence		245.8	228.8
	ClusterTendency		166.4	150.1
	Contrast		139.2	131.2
	DifferenceAverage		83.4	76.7
	DifferenceEntropy		43.9	40.0
	DifferenceVariance		128.7	122.4
	Imc1		70.1	61.6
	Imc2		32.6	25.1
	JointEnergy		102.5	86.9
	JointEntropy		38.5	30.2
	MaximumProbability		99.3	87.1
	MCC		39.7	33.9
	SumEntropy		37.1	29.8
	SumSquares		157.4	142.9
**gldm**	DependenceEntropy		12.3	11.3
	GrayLevelNonUniformity		75.0	66.1
	GrayLevelVariance		157.0	140.6
	SmallDependenceEmphasis		81.3	72.1
	SmallDependenceHighGrayLevelEmphasis		164.1	141.4
**glrlm**	GrayLevelNonUniformityNormalized		67.7	60.5

	SumSquares		69.8	43.1
**gldm**	DependenceNonUniformity		17.2	10.1
	DependenceNonUniformityNormalized		17.2	10.1
	DependenceVariance		36.6	20.3
	GrayLevelNonUniformity		27.1	16.2
	GrayLevelVariance		70.3	43.4
	LargeDependenceEmphasis		37.8	21.3
	SmallDependenceEmphasis		32.5	16.9
	SmallDependenceHighGrayLevelEmphasis		80.9	47.6
**glrlm**	GrayLevelNonUniformity		18.1	11.3
	GrayLevelNonUniformityNormalized		25.8	15.1
	GrayLevelVariance		67.2	41.9
	LongRunEmphasis		21.1	10.9
	LongRunHighGrayLevelEmphasis		59.8	39.8
	RunEntropy		6.3	4.1
	RunLengthNonUniformity		19.5	9.5
	RunLengthNonUniformityNormalized		12.5	6.2
	RunPercentage		7.6	3.8
	RunVariance		42.8	25.6
	ShortRunEmphasis		6.5	3.1
	ShortRunHighGrayLevelEmphasis		63.5	42.2
**ngtdm**	Busyness		55.0	35.0
	Complexity		84.2	53.6
	Contrast		43.2	30.9
	Strength		59.7	35.0

tissue

			Whole gland
**glcm**	InverseVariance	benign	9.8
		tumor	19.3

	GrayLevelVariance	151.9	135.9
	LongRunEmphasis	65.9	54.0
	RunEntropy	20.0	17.9
	RunLengthNonUniformityNormalized	36.8	31.1
	RunPercentage	24.5	19.5
	ShortRunEmphasis	21.9	16.8
	ShortRunHighGrayLevelEmphasis	137.6	119.5
**ngtdm**	Busyness	150.3	134.9
	Complexity	172.5	159.9
	Strength	154.8	135.0

whole gland

		Whole gland
**glcm**	Autocorrelation	140.2
	Correlation	140.2
	Id	130.5
	Idm	130.5
	Idmn	1.5
	Idn	4.9
	InverseVariance	45.9
	JointAverage	78.9
	SumAverage	78.9
**gldm**	DependenceNonUniformity	361.6
	DependenceNonUniformityNormalized	55.0
	DependenceVariance	125.2
	HighGrayLevelEmphasis	131.5
	LargeDependenceEmphasis	99.4
	LargeDependenceHighGrayLevelEmphasis	145.7
	LargeDependenceLowGrayLevelEmphasis	203.5
	LowGrayLevelEmphasis	203.5
	SmallDependenceLowGrayLevelEmphasis	155.4

whole gland

		Whole gland
**glcm**	Autocorrelation	45.4
	ClusterShade	115.8
	JointAverage	23.8
	SumAverage	23.8
**gldm**	HighGrayLevelEmphasis	43.2
	LargeDependenceHighGrayLevelEmphasis	47.0
	LargeDependenceLowGrayLevelEmphasis	76.6
	LowGrayLevelEmphasis	69.3
	SmallDependenceLowGrayLevelEmphasis	68.3
**glrlm**	HighGrayLevelRunEmphasis	41.5
	LongRunLowGrayLevelEmphasis	71.0
	LowGrayLevelRunEmphasis	68.6
	ShortRunLowGrayLevelEmphasis	68.8
**ngtdm**	Coarseness	6.0

**glrlm**	GrayLevelNonUniformity	353.8
	HighGrayLevelRunEmphasis	126.6
	LongRunHighGrayLevelEmphasis	124.0
	LongRunLowGrayLevelEmphasis	187.7
	LowGrayLevelRunEmphasis	166.5
	RunLengthNonUniformity	354.6
	RunVariance	118.0
	ShortRunLowGrayLevelEmphasis	160.8
**ngtdm**	Coarseness	54.6
	Contrast	108.9

GLCM - grey level co-occurrence matrix; GLRLM - grey level run length matrix; GLDM - grey level dependence matrix; NGTDM - neighboring grey tone difference matrix.

Table S3-7 Repeatability coefficients (%RC) of region of interest (ROI) and voxel-wise measurements of radiomic features derived from HS map stratified by their dependency to tissue type (benign, B, and tumor, T) and anatomical zone (peripheral zone, PZ, and non-peripheral zone, nPZ).

			$\%RC_{ROI}$	
**tissue and zone**				
			PZ	nPZ
**glcm**	Correlation	benign	11.8	5.2
		tumor	10.9	16.6
**zone**				
			PZ	nPZ
**glcm**	InverseVariance		10.8	7.5
**gldm**	LargeDependenceLowGrayLevelEmphasis		29.7	18.4
	SmallDependenceEmphasis		30.8	22.8
	SmallDependenceHighGrayLevelEmphasis		33.8	20.9
**glrlm**	LongRunEmphasis		24.9	18.3
	LongRunLowGrayLevelEmphasis		28.0	17.6
	ShortRunEmphasis		11.4	8.0
	ShortRunHighGrayLevelEmphasis		23.8	16.2
**tissue**				
			Whole gland	
**glcm**	Imc1	benign	7.9	
		tumor	16.3	
**glrlm**	GrayLevelNonUniformity	benign	4.5	
		tumor	9.4	
	RunVariance	benign	15.7	
		tumor	27.3	
**ngtdm**	Coarseness	benign	2.8	
		tumor	8.0	

			Median $\%RC_{voxel}$	
**tissue and zone**				
			PZ	nPZ
**glcm**	Imc2	benign	57.2	55.3
		tumor	42.8	51.8
	JointEntropy	benign	56.8	53.4
		tumor	42.9	49.1
	MCC	benign	58.8	57.3
		tumor	44.5	53.9
	SumEntropy	benign	53.0	50.1
		tumor	40.8	46.3
**ngtdm**	Coarseness	benign	69.1	59.8
		tumor	47.8	52.8
**zone**				
			PZ	nPZ
**glcm**	ClusterProminence		225.8	213.9
	ClusterTendency		149.3	140.9
	DifferenceAverage		80.5	75.6
	DifferenceEntropy		43.6	41.6
	InverseVariance		58.2	53.0
	JointEnergy		102.6	99.8
	MaximumProbability		91.7	89.5
	SumSquares		137.1	129.0
**gldm**	DependenceEntropy		16.0	14.8
	GrayLevelNonUniformity		66.8	63.5
	GrayLevelVariance		136.2	127.4
	SmallDependenceEmphasis		83.3	79.0

whole gland

		Whole gland
**glcm**	Autocorrelation	20.8
	ClusterProminence	88.8
	ClusterShade	1018.0
	ClusterTendency	43.5
	Contrast	34.1
	DifferenceAverage	21.9
	DifferenceEntropy	12.9
	DifferenceVariance	24.5
	Id	6.1
	Idm	7.0
	Idmn	0.1
	Idn	0.6
	Imc2	10.4
	JointAverage	10.5
	JointEnergy	25.7
	JointEntropy	13.7
	MaximumProbability	20.9
	MCC	9.6
	SumAverage	10.5
	SumEntropy	11.4
	SumSquares	40.8
**gldm**	DependenceEntropy	2.3
	DependenceNonUniformity	5.9
	DependenceNonUniformityNormalized	5.9
	DependenceVariance	13.8
	GrayLevelNonUniformity	16.4
	GrayLevelVariance	41.6
	HighGrayLevelEmphasis	20.6
	LargeDependenceEmphasis	20.9

**glrlm**	GrayLevelNonUniformityNormalized	58.4	54.6
	GrayLevelVariance	123.8	114.1
	LongRunEmphasis	76.4	72.9
	LongRunHighGrayLevelEmphasis	86.2	80.9
	RunEntropy	20.5	18.2
	RunLengthNonUniformityNormalized	42.3	41.0
	RunPercentage	33.7	31.6
	ShortRunEmphasis	36.2	32.7
	ShortRunHighGrayLevelEmphasis	50.7	44.6
**ngtdm**	Busyness	156.9	146.6
	Complexity	155.2	146.1
	Contrast	88.4	81.6
	Strength	141.9	131.9

tissue

			Whole gland
**glcm**	Id	benign	66.3
		tumor	74.6
	Idm	benign	66.3
		tumor	74.6
	Imc1	benign	81.9
		tumor	74.4
**gldm**	DependenceNonUniformityNormalized	benign	44.5
		tumor	46.3
	DependenceVariance	benign	97.6
		tumor	100.9
**glrlm**	GrayLevelNonUniformity		691.3
	RunLengthNonUniformity	benign	346.0
		tumor	349.3

whole gland

	LargeDependenceHighGrayLevelEmphasis	35.5
	LowGrayLevelEmphasis	21.9
	SmallDependenceLowGrayLevelEmphasis	40.1
**glrlm**	GrayLevelNonUniformityNormalized	15.7
	GrayLevelVariance	38.0
	HighGrayLevelRunEmphasis	20.5
	LongRunHighGrayLevelEmphasis	34.3
	LowGrayLevelRunEmphasis	21.9
	RunEntropy	3.8
	RunLengthNonUniformity	19.6
	RunLengthNonUniformityNormalized	11.3
	RunPercentage	8.6
	ShortRunLowGrayLevelEmphasis	26.8
**ngtdm**	Busyness	18.5
	Complexity	50.0
	Contrast	14.0
	Strength	40.3

		Whole gland
**glcm**	Autocorrelation	39.9
	ClusterShade	88902.9
	Contrast	109.1
	Correlation	39.9
	DifferenceVariance	81.8
	Idmn	0.3
	Idn	2.2
	JointAverage	20.5
	SumAverage	20.5
**gldm**	DependenceNonUniformity	361.6
	HighGrayLevelEmphasis	38.8
	LargeDependenceEmphasis	82.3
	LargeDependenceHighGrayLevelEmphasis	91.6
	LargeDependenceLowGrayLevelEmphasis	85.6
	LowGrayLevelEmphasis	85.6
	SmallDependenceHighGrayLevelEmphasis	85.7
	SmallDependenceLowGrayLevelEmphasis	94.4
**glrlm**	HighGrayLevelRunEmphasis	38.1
	LongRunLowGrayLevelEmphasis	79.8
	LowGrayLevelRunEmphasis	44.1
	RunVariance	94.4
	ShortRunLowGrayLevelEmphasis	55.0

GLCM - grey level co-occurrence matrix; GLRLM - grey level run length matrix; GLDM - grey level dependence matrix; NGTDM - neighboring grey tone difference matrix.

Table S3-8 Repeatability coefficients (%RC) of region of interest (ROI) and voxel-wise measurements of radiomic features derived from R2 map stratified by their dependency to tissue type (benign, B, and tumor, T) and anatomical zone (peripheral zone, PZ, and non-peripheral zone, nPZ).*

	**$\%RC_{ROI}$**	
**whole gland**		
		Whole gland
**glcm**	Autocorrelation	35.4
	ClusterProminence	386.4
	ClusterShade	452.3
	ClusterTendency	383.2
	Contrast	378.9
	Correlation	51.9
	DifferenceAverage	378.9
	DifferenceEntropy	348.9
	DifferenceVariance	377.7
	Id	3.4
	Idm	3.4
	Idmn	0.0
	Idn	0.3
	Imc1	389.1
	Imc2	383.8
	InverseVariance	378.9
	JointAverage	21.3
	JointEnergy	17.1
	JointEntropy	332.0
	MaximumProbability	9.9
	MCC	386.4
	SumAverage	21.3
	SumEntropy	344.6
	SumSquares	381.4
**gldm**	DependenceEntropy	31.4

	**Median $\%RC_{voxel}$**			
**zone**				
			PZ	nPZ
**glcm**	ClusterProminence		338.7	346.8
	ClusterTendency		335.9	345.5
	Contrast		326.5	339.7
	DifferenceAverage		325.6	339.7
	DifferenceVariance		323.7	337.8
	SumSquares		332.3	343.1
**gldm**	GrayLevelVariance		329.4	340.7
**glrlm**	GrayLevelNonUniformity		301.9	298.6
	GrayLevelVariance		319.6	334.8
	RunLengthNonUniformity		288.6	283.0
**ngtdm**	Complexity		326.4	339.4
	Contrast		339.2	348.2
	Strength		310.9	329.3
**tissue**				
			Whole gland	
**glcm**	DifferenceEntropy	benign	541.0	
		tumor	281.3	
	Idmn	benign	13.9	
		tumor	2.6	
	Idn	benign	14.8	
		tumor	3.0	
	JointEnergy	benign	79.6	
		tumor	65.1	
	MaximumProbability	benign	58.2	

	DependenceNonUniformity	23.1
	DependenceNonUniformityNormalized	23.1
	DependenceVariance	2.8
	GrayLevelNonUniformity	11.7
	GrayLevelVariance	377.9
	HighGrayLevelEmphasis	35.2
	LargeDependenceEmphasis	9.5
	LargeDependenceHighGrayLevelEmphasis	39.6
	LargeDependenceLowGrayLevelEmphasis	62.3
	LowGrayLevelEmphasis	56.7
	SmallDependenceEmphasis	39.4
	SmallDependenceHighGrayLevelEmphasis	36.0
	SmallDependenceLowGrayLevelEmphasis	91.0
**glrlm**	GrayLevelNonUniformity	5.1
	GrayLevelNonUniformityNormalized	21.0
	GrayLevelVariance	373.8
	HighGrayLevelRunEmphasis	36.7
	LongRunEmphasis	22.6
	LongRunHighGrayLevelEmphasis	51.4
	LongRunLowGrayLevelEmphasis	63.9
	LowGrayLevelRunEmphasis	57.7
	RunEntropy	33.9
	RunLengthNonUniformity	0.1
	RunLengthNonUniformityNormalized	24.7
	RunPercentage	14.5
	RunVariance	29.3
	ShortRunEmphasis	42.6
	ShortRunHighGrayLevelEmphasis	36.8
	ShortRunLowGrayLevelEmphasis	94.5
**ngtdm**	Busyness	381.0
	Coarseness	128.8

		tumor	45.3
	SumEntropy	benign	449.8
		tumor	286.1
**gldm**	DependenceEntropy	benign	67.4
		tumor	55.2
	DependenceNonUniformityNormalized	benign	63.9
		tumor	54.7
	LargeDependenceEmphasis	benign	53.7
		tumor	38.2
	LowGrayLevelEmphasis	benign	59.3
		tumor	42.2
	SmallDependenceLowGrayLevelEmphasis	benign	115.7
		tumor	101.0
**glrlm**	GrayLevelNonUniformityNormalized	benign	66.3
		tumor	50.1
	LongRunEmphasis	benign	77.4
		tumor	71.1
	LowGrayLevelRunEmphasis	benign	64.6
		tumor	49.3
	RunEntropy	benign	67.4
		tumor	56.3
	RunLengthNonUniformityNormalized	benign	61.9
		tumor	56.6
	RunPercentage	benign	46.2
		tumor	40.9
	RunVariance	benign	76.6
		tumor	74.4
	ShortRunEmphasis	benign	85.2
		tumor	73.5
	ShortRunLowGrayLevelEmphasis	benign	113.7
		tumor	94.2

	Complexity	378.1
	Contrast	384.5
	Strength	354.8

whole gland

		Whole gland
**glcm**	Autocorrelation	54.3
	ClusterShade	22466.3
	Correlation	54.3
	Id	12.2
	Idm	12.2
	Imc1	340.1
	Imc2	334.9
	InverseVariance	330.7
	JointAverage	41.1
	JointEntropy	395.0
	MCC	336.9
	SumAverage	41.1
**gldm**	DependenceNonUniformity	361.6
	DependenceVariance	39.7
	GrayLevelNonUniformity	50.0
	HighGrayLevelEmphasis	51.6
	LargeDependenceHighGrayLevelEmphasis	68.7
	LargeDependenceLowGrayLevelEmphasis	60.0
	SmallDependenceEmphasis	83.2
	SmallDependenceHighGrayLevelEmphasis	91.5
**glrlm**	HighGrayLevelRunEmphasis	50.4
	LongRunHighGrayLevelEmphasis	90.7
	LongRunLowGrayLevelEmphasis	69.9
	ShortRunHighGrayLevelEmphasis	89.5
**ngtdm**	Busyness	353.1
	Coarseness	216.0

GLCM - grey level co-occurrence matrix; GLRLM - grey level run length matrix; GLDM - grey level dependence matrix; NGTDM - neighboring grey tone difference matrix.

Table S3-9 Repeatability coefficients (%RC) of region of interest (ROI) and voxel-wise measurements of radiomic features derived from T1 map stratified by their dependency to tissue type (benign, B, and tumor, T) and anatomical zone (peripheral zone, PZ, and non-peripheral zone, nPZ).

	$\%RC_{ROI}$			
**tissue and zone**				
			PZ	nPZ
**glrlm**	GrayLevelNonUniformity	benign	9.7	4.5
		tumor	20.5	7.4
**zone**				
			PZ	nPZ
**glcm**	Idmn		0.1	0.1
	Idn		1.1	0.5
**ngtdm**	Coarseness		7.5	4.3
	Contrast		24.2	12.6
**whole gland**				
			Whole gland	
**glcm**	Autocorrelation		26.0	
	ClusterProminence		80.2	
	ClusterShade		75.7	
	ClusterTendency		39.4	
	Contrast		35.7	
	Correlation		8.0	
	DifferenceAverage		21.0	
	DifferenceEntropy		12.0	
	DifferenceVariance		26.9	
	Id		6.8	
	Idm		8.3	
	Imc1		12.4	
	Imc2		9.2	

	Median $\%RC_{voxel}$			
**tissue and zone**				
			PZ	nPZ
**glcm**	Id	benign	102.8	97.0
		tumor	93.6	91.0
	Idm	benign	102.8	97.0
		tumor	93.6	91.0
**zone**				
			PZ	nPZ
**glcm**	Contrast	all	114.8	103.2
	DifferenceVariance	all	96.4	84.5
**gldm**	DependenceNonUniformityNormalized	all	45.6	45.6
	DependenceVariance	all	100.1	99.3
**tissue**				
**glrlm**	ShortRunHighGrayLevelEmphasis	benign	57.2	
		tumor	51.5	
**whole gland**				
			Whole gland	
**glcm**	Autocorrelation		42.0	
	ClusterProminence		223.9	
	ClusterShade		96179.0	
	ClusterTendency		139.5	
	Correlation		42.0	
	DifferenceAverage		73.4	

	InverseVariance	4.5
	JointAverage	13.0
	JointEnergy	24.5
	JointEntropy	11.6
	MaximumProbability	20.8
	MCC	8.3
	SumAverage	13.0
	SumEntropy	9.2
	SumSquares	38.3
**gldm**	DependenceEntropy	1.6
	DependenceNonUniformity	8.6
	DependenceNonUniformityNormalized	8.6
	DependenceVariance	18.1
	GrayLevelNonUniformity	15.1
	GrayLevelVariance	37.8
	HighGrayLevelEmphasis	26.0
	LargeDependenceEmphasis	21.9
	LargeDependenceHighGrayLevelEmphasis	23.3
	LargeDependenceLowGrayLevelEmphasis	45.9
	LowGrayLevelEmphasis	26.1
	SmallDependenceEmphasis	23.1
	SmallDependenceHighGrayLevelEmphasis	46.9
	SmallDependenceLowGrayLevelEmphasis	21.5
**glrlm**	GrayLevelNonUniformityNormalized	13.7
	GrayLevelVariance	34.9
	HighGrayLevelRunEmphasis	25.8
	LongRunEmphasis	17.2
	LongRunHighGrayLevelEmphasis	22.1
	LongRunLowGrayLevelEmphasis	41.7
	LowGrayLevelRunEmphasis	26.0
	RunEntropy	3.1

	DifferenceEntropy	41.3
	Idmn	0.5
	Idn	2.4
	Imc1	80.1
	Imc2	50.7
	InverseVariance	42.4
	JointAverage	21.5
	JointEnergy	92.4
	JointEntropy	47.7
	MaximumProbability	84.1
	MCC	55.2
	SumAverage	21.5
	SumEntropy	43.6
	SumSquares	128.3
**gldm**	DependenceEntropy	13.1
	DependenceNonUniformity	361.6
	GrayLevelNonUniformity	59.5
	GrayLevelVariance	127.2
	HighGrayLevelEmphasis	40.6
	LargeDependenceEmphasis	78.3
	LargeDependenceHighGrayLevelEmphasis	82.6
	LargeDependenceLowGrayLevelEmphasis	95.0
	LowGrayLevelEmphasis	95.0
	SmallDependenceEmphasis	74.0
	SmallDependenceHighGrayLevelEmphasis	93.1
	SmallDependenceLowGrayLevelEmphasis	79.1
**glrlm**	GrayLevelNonUniformity	347.7
	GrayLevelNonUniformityNormalized	51.8
	GrayLevelVariance	118.0
	HighGrayLevelRunEmphasis	40.2
	LongRunEmphasis	65.8

	RunLengthNonUniformity	16.8
	RunLengthNonUniformityNormalized	10.3
	RunPercentage	6.9
	RunVariance	25.1
	ShortRunEmphasis	6.3
	ShortRunHighGrayLevelEmphasis	31.2
	ShortRunLowGrayLevelEmphasis	23.6
**ngtdm**	Busyness	27.8
	Complexity	50.8
	Strength	33.3

	LongRunHighGrayLevelEmphasis	70.2
	LongRunLowGrayLevelEmphasis	83.3
	LowGrayLevelRunEmphasis	44.8
	RunEntropy	17.0
	RunLengthNonUniformity	348.9
	RunLengthNonUniformityNormalized	36.9
	RunPercentage	27.7
	RunVariance	94.4
	ShortRunEmphasis	27.1
	ShortRunLowGrayLevelEmphasis	47.3
**ngtdm**	Busyness	130.7
	Coarseness	52.3
	Complexity	149.9
	Contrast	89.5
	Strength	132.8

GLCM - grey level co-occurrence matrix; GLRLM - grey level run length matrix; GLDM - grey level dependence matrix; NGTDM - neighboring grey tone difference matrix.

Supplementary Data 4

Table S4-1 Top 50 most repeatable radiomics features measured using ROI approach. Features are sorted by the image/map it was extracted from, feature class, and feature name. The threshold category indicates whether the measurement uncertainty is dependent on both tissue and region, tissue only, anatomical region only, or independent of tissue and region (whole gland). Highlighted features indicate that the same feature was ranked in the top 50 in both ROI and voxel-wise measurement approaches.

Feature class	Feature name	Image type	Threshold category
glcm	Idmn	2dt2w	whole gland
glcm	Idn	2dt2w	whole gland
glcm	Imc2	2dt2w	whole gland
glcm	InverseVariance	2dt2w	whole gland
glcm	JointEntropy	2dt2w	whole gland
glcm	MCC	2dt2w	whole gland
glcm	SumEntropy	2dt2w	whole gland
gldm	DependenceEntropy	2dt2w	tissue
glrlm	RunEntropy	2dt2w	whole gland
glrlm	RunLengthNonUniformityNormalized	2dt2w	whole gland
glrlm	RunPercentage	2dt2w	whole gland
glrlm	ShortRunEmphasis	2dt2w	whole gland
glcm	Idmn	adc_50_800	whole gland
glcm	Idn	adc_50_800	whole gland
glcm	InverseVariance	adc_50_800	whole gland
gldm	DependenceEntropy	adc_50_800	zone
glrlm	RunEntropy	adc_50_800	zone
glcm	Idmn	d	whole gland
glcm	Idn	d	whole gland
gldm	DependenceEntropy	d	whole gland
glrlm	RunEntropy	d	whole gland
glcm	Idmn	f	zone
glcm	Idn	f	zone
gldm	DependenceEntropy	f	tissue and zone
glrlm	RunEntropy	f	zone

glrlm	ShortRunEmphasis	f	zone
ngtdm	Coarseness	f	whole gland
glcm	Id	hs	whole gland
glcm	Idm	hs	whole gland
glcm	Idmn	hs	whole gland
glcm	Idn	hs	whole gland
gldm	DependenceEntropy	hs	whole gland
gldm	DependenceNonUniformity	hs	whole gland
gldm	DependenceNonUniformityNormalized	hs	whole gland
glrlm	RunEntropy	hs	whole gland
glcm	Id	r2star	whole gland
glcm	Idm	r2star	whole gland
glcm	Idmn	r2star	whole gland
glcm	Idn	r2star	whole gland
gldm	DependenceVariance	r2star	whole gland
glrlm	GrayLevelNonUniformity	r2star	whole gland
glrlm	RunLengthNonUniformity	r2star	whole gland
glcm	Id	t1map	whole gland
glcm	Idmn	t1map	zone
glcm	Idn	t1map	zone
glcm	InverseVariance	t1map	whole gland
gldm	DependenceEntropy	t1map	whole gland
glrlm	RunEntropy	t1map	whole gland
glrlm	RunPercentage	t1map	whole gland
glrlm	ShortRunEmphasis	t1map	whole gland

GLCM - grey level co-occurrence matrix; GLRLM - grey level run length matrix; GLDM - grey level dependence matrix; NGTDM - neighboring grey tone difference matrix.

Table S4- 2 Top 50 most repeatable radiomics features measured using voxel-wise approach. Features are sorted by the image/map it was extracted from, feature class, and feature name. The threshold category indicates whether the measurement uncertainty is dependent on both tissue and region, tissue only, anatomical region only, or independent of tissue and region (whole gland). Highlighted features indicate that the same feature was ranked in the top 50 in both ROI and voxel-wise measurement approaches.

Feature class	Feature name	Image type	Threshold category
glcm	DifferenceEntropy	2dt2w	whole gland
glcm	Idmn	2dt2w	whole gland
glcm	Idn	2dt2w	tissue
glcm	Imc2	2dt2w	whole gland
glcm	JointEntropy	2dt2w	whole gland
glcm	SumAverage	2dt2w	whole gland
glcm	SumEntropy	2dt2w	whole gland
gldm	DependenceEntropy	2dt2w	whole gland
glrlm	RunEntropy	2dt2w	whole gland
glrlm	RunLengthNonUniformityNormalized	2dt2w	whole gland
glrlm	RunPercentage	2dt2w	whole gland
glrlm	ShortRunEmphasis	2dt2w	whole gland
glcm	Idmn	adc_50_800	whole gland
glcm	Idn	adc_50_800	whole gland
gldm	DependenceEntropy	adc_50_800	whole gland
glrlm	RunEntropy	adc_50_800	zone
glcm	DifferenceEntropy	d	whole gland
glcm	Idmn	d	whole gland
glcm	Idn	d	tissue
glcm	Imc2	d	whole gland
glcm	JointEntropy	d	whole gland
glcm	SumEntropy	d	whole gland
gldm	DependenceEntropy	d	whole gland
glrlm	RunEntropy	d	whole gland
glrlm	RunLengthNonUniformityNormalized	d	whole gland
glrlm	RunPercentage	d	whole gland
glrlm	ShortRunEmphasis	d	whole gland
glcm	Idmn	f	whole gland
glcm	Idn	f	whole gland
gldm	DependenceEntropy	f	zone
glrlm	RunEntropy	f	zone
glrlm	RunPercentage	f	zone
glrlm	ShortRunEmphasis	f	zone

glcm	Idmn	hs	whole gland
glcm	Idn	hs	whole gland
glcm	JointAverage	hs	whole gland
glcm	SumAverage	hs	whole gland
gldm	DependenceEntropy	hs	zone
glrlm	RunEntropy	hs	zone
glcm	Id	r2star	whole gland
glcm	Idm	r2star	whole gland
glcm	Idmn	r2star	tissue
glcm	Idn	r2star	tissue
glcm	Idmn	t1map	whole gland
glcm	Idn	t1map	whole gland
glcm	JointAverage	t1map	whole gland
glcm	SumAverage	t1map	whole gland
gldm	DependenceEntropy	t1map	whole gland
glrlm	RunEntropy	t1map	whole gland
glrlm	ShortRunEmphasis	t1map	whole gland

GLCM - grey level co-occurrence matrix; GLRLM - grey level run length matrix; GLDM - grey level dependence matrix; NGTDM - neighboring grey tone difference matrix.

Table S4 Top 50 most repeatable radiomics features measured using ROI approach. Features are sorted by the image/map it was extracted from, feature class, and feature name. The threshold category indicates whether the measurement uncertainty is dependent on both tissue and region, tissue only, anatomical region only, or independent of tissue and region (whole gland). Highlighted features indicate that the same feature was ranked in the top 50 in both ROI and voxel-wise measurement approaches.

Feature class	Feature name	Image type	Threshold category
glcm	Idmn	2dt2w	whole gland
glcm	Idn	2dt2w	whole gland
glcm	Imc2	2dt2w	whole gland
glcm	InverseVariance	2dt2w	whole gland
glcm	JointEntropy	2dt2w	whole gland
glcm	MCC	2dt2w	whole gland
glcm	SumEntropy	2dt2w	whole gland
gldm	DependenceEntropy	2dt2w	tissue
glrlm	RunEntropy	2dt2w	whole gland

glrlm	RunLengthNonUniformityNormalized	2dt2w	whole gland
glrlm	RunPercentage	2dt2w	whole gland
glrlm	ShortRunEmphasis	2dt2w	whole gland
glcm	Idmn	adc_50_800	whole gland
glcm	Idn	adc_50_800	whole gland
glcm	InverseVariance	adc_50_800	whole gland
gldm	DependenceEntropy	adc_50_800	zone
glrlm	RunEntropy	adc_50_800	zone
glcm	Idmn	d	whole gland
glcm	Idn	d	whole gland
gldm	DependenceEntropy	d	whole gland
glrlm	RunEntropy	d	whole gland
glcm	Idmn	f	zone
glcm	Idn	f	zone
gldm	DependenceEntropy	f	tissue and zone
glrlm	RunEntropy	f	zone
glrlm	ShortRunEmphasis	f	zone
ngtdm	Coarseness	f	whole gland
glcm	Id	hs	whole gland
glcm	Idm	hs	whole gland
glcm	Idmn	hs	whole gland
glcm	Idn	hs	whole gland
gldm	DependenceEntropy	hs	whole gland
gldm	DependenceNonUniformity	hs	whole gland
gldm	DependenceNonUniformityNormalized	hs	whole gland
glrlm	RunEntropy	hs	whole gland
glcm	Id	r2star	whole gland
glcm	Idm	r2star	whole gland
glcm	Idmn	r2star	whole gland
glcm	Idn	r2star	whole gland
gldm	DependenceVariance	r2star	whole gland
glrlm	GrayLevelNonUniformity	r2star	whole gland
glrlm	RunLengthNonUniformity	r2star	whole gland

glcm	Id	t1map	whole gland
glcm	Idmn	t1map	zone
glcm	Idn	t1map	zone
glcm	InverseVariance	t1map	whole gland
gldm	DependenceEntropy	t1map	whole gland
glrlm	RunEntropy	t1map	whole gland
glrlm	RunPercentage	t1map	whole gland
glrlm	ShortRunEmphasis	t1map	whole gland

GLCM - grey level co-occurrence matrix; GLRLM - grey level run length matrix; GLDM - grey level dependence matrix; NGTDM - neighboring grey tone difference matrix.
